# Review on a Traditional Herbal Medicine, *Eurycoma longifolia* Jack (Tongkat Ali): Its Traditional Uses, Chemistry, Evidence-Based Pharmacology and Toxicology

**DOI:** 10.3390/molecules21030331

**Published:** 2016-03-10

**Authors:** Shaheed Ur Rehman, Kevin Choe, Hye Hyun Yoo

**Affiliations:** Institute of Pharmaceutical Science and Technology and College of Pharmacy, Hanyang University, Ansan, Gyeonggi-do 426-791, Korea; dr.shaheedmarwat@yahoo.com (S.U.R.); kkchoe@hanyang.ac.kr (K.C.)

**Keywords:** traditional herbal medicine, *Eurycoma longifolia*, quassinoids, pharmacological effects, safety

## Abstract

*Eurycoma longifolia* Jack (known as tongkat ali), a popular traditional herbal medicine, is a flowering plant of the family Simaroubaceae, native to Indonesia, Malaysia, Vietnam and also Cambodia, Myanmar, Laos and Thailand. *E. longifolia*, is one of the well-known folk medicines for aphrodisiac effects as well as intermittent fever (malaria) in Asia. Decoctions of *E. longifolia* leaves are used for washing itches, while its fruits are used in curing dysentery. Its bark is mostly used as a vermifuge, while the taproots are used to treat high blood pressure, and the root bark is used for the treatment of diarrhea and fever. Mostly, the roots extract of *E. longifolia* are used as folk medicine for sexual dysfunction, aging, malaria, cancer, diabetes, anxiety, aches, constipation, exercise recovery, fever, increased energy, increased strength, leukemia, osteoporosis, stress, syphilis and glandular swelling. The roots are also used as an aphrodisiac, antibiotic, appetite stimulant and health supplement. The plant is reported to be rich in various classes of bioactive compounds such as quassinoids, canthin-6-one alkaloids, β-carboline alkaloids, triterpene tirucallane type, squalene derivatives and biphenyl neolignan, eurycolactone, laurycolactone, and eurycomalactone, and bioactive steroids. Among these phytoconstituents, quassinoids account for a major portion of the *E*. *longifolia* root phytochemicals. An acute toxicity study has found that the oral Lethal Dose 50 (LD_50_) of the alcoholic extract of *E. longifolia* in mice is between 1500–2000 mg/kg, while the oral LD_50_ of the aqueous extract form is more than 3000 mg/kg. Liver and renal function tests showed no adverse changes at normal daily dose and chronic use of *E. longifolia*. Based on established literature on health benefits of *E. longifolia*, it is important to focus attention on its more active constituents and the constituents’ identification, determination, further development and most importantly, the standardization. Besides the available data, more evidence is required regarding its therapeutic efficacy and safety, so it can be considered a rich herbal source of new drug candidates. It is very important to conserve this valuable medicinal plant for the health benefit of future generations.

## 1. Introduction

### 1.1. Traditional, Complementary/Alternative and Herbal Medicine

#### 1.1.1. Traditional Medicine

This is also well known as indigenous or folk medicine. According to the World Health Organization (WHO), traditional medicine is defined as “the sum total of the knowledge, skills, and practices based on the theories, beliefs, and experiences indigenous to different cultures, whether explicable or not, used in the maintenance of health as well as in the prevention, diagnosis, improvement or treatment of physical and mental illness” [1].

#### 1.1.2. Complementary/Alternative Medicine

The terms “complementary medicine” and/or “alternative medicine” (and sometimes also “non-conventional”) are used interchangeably with “traditional medicine” in some countries. Complementary/alternative medicine refers to a broad set of health care and health-related practices that are not part of that specific country's own tradition, and are not considered the dominant health care system [2].

#### 1.1.3. Herbal Medicine

According to the WHO, it includes herbs, herbal preparations, herbal materials, and all finished herbal products, that contain plants, other plant materials, or combinations, as an active ingredient [2].

#### 1.1.4. Traditional Use of Herbal Medicines

This refers to the long historic or traditional use of herbal based medicines. The uses of these medicines are well-established and widely acknowledged their safety and efficacy, as well as accepted by national health authorities [2]. Traditionally employed, indigenous herbal or herb-derived medicines have been very popular from time immemorial; and today, these medicines have commanded much attention worldwide, due to their natural origin and nutraceutical potential [2,3]. The World Health Organization has estimated that 80% of people worldwide rely on herbal medicines for some part of their primary health care needs [4]. When adopted outside of its traditional culture, traditional medicine is often called complementary and alternative medicine [1].

Worldwide, many traditional medicine systems (TMS) are used, including Chinese Traditional Medicine, Indian Ayurvedic Medicine, and the popular Unani Medicine of Arab cultures. Many other indigenous traditional medicine systems have also been developed in past history by African, Asian, Arabic, Pacific, American, and also some other cultures. The theory and application of these traditional medicine systems, differ significantly from those of well-developed allopathic medicines [3]. Today, the increasing demands of use of traditional herbal therapies, more likely based on the good past experiences of the effectiveness as well as safety of these herbal medicines, still require positive research evidence, so recent developments in the biological and analytical sciences, along with innovations in proteomics and genomics surely can play a dominant role in the validation of traditionally based herbal medicines, to further improve their quality, safety and efficacy with clinic-based evidence [5,6].

### 1.2. Eurycoma longifolia Jack—A Promising Herbal Medicine

This is a well justified fact that the traditional medicines as well as complementary and alternative medicines have the well-established role in our health. *E. longifolia* Jack (Tongkat Ali) is one of the most well-known herbal folk medicines in Southeast Asia. Its roots are traditionally used for many disorders and diseases, in many countries Asia. Besides this, recently *E. longifolia* has contributed good role as a complementary and alternative medicine in herbal therapies, in the West.

#### 1.2.1. Synonyms

Tongkat Ali, Ali’s Umbrella or Malaysia ginseng (Malaysia), Pasak Bumi or Bedara Pahit (Indonesia), Ian-don (Thailand), and Cay ba benh (Vietnam), tho nan (Laotian).

#### 1.2.2. Origin

Indigenous to South-East Asian countries like Malaysia, Indonesia, and Vietnam, some of the plant species are also found in certain patches in regions of Cambodia, Myanmar, Laos and in Thailand [5,7,8,9,10,11]. It is planted mainly in Malaysia for its medicinal value in order to conserve the wild plants [12,13,14,15].

Besides *Eurycoma longifolia* Jack, there are three other plant species also known locally as Tongkat Ali, which literally means “Ali’s walking stick,” which refers to its aphrodisiac property. Some authors claim it gets its name “stick” from the long twisted roots that are harvested for their medicinal value. The three plant species are *Entomophthora apiculata,* *Polyathia bullata,* and *Goniothalamus* sp. [16].

“Malaysian ginseng” as it is known in Malaysia, is also regarded as an adaptogen [17], an herb or herbal compound that assists in combating stress and disease and improves physical strength without adverse effects.

#### 1.2.3. Description

*E. longifolia* is a tall, slender, shrubby tree, which grows in sandy soil. It belongs to the Simaroubaceae family. It has compound leaves on branches that can grow up to 1 m long. The leaves are pinnate in shape and green in colour. The numerous leaflets are opposite or subopposite, lanceolate to ovate-lanceolate, 5–20 cm by 1.5–6 cm, with smooth margins. Flowers are tiny, reddish, unisexual and are densely arranged. The drupes are ovoid with a distinct ridge, 1–2 cm by 0.5–1.2 cm and they turn dark reddish brown when ripe [18,19,20].

### 1.3. Genetic Diversity

The genetic diversity of *E. longifolia* is decreasing due to widespread harvesting; thus, single nucleotide polymorphisms have been used to study the remaining diversity [21], and microsatellite markers have been studied as tools for DNA profiling and genetic diversity studies [22]. Razi *et al.*, showed that in an uncontrolled cultivated area, the *E. longifolia* samples could be characterized based on their cultivar’s origins. They proved that identification of *E. longifolia* from various cultivars can be obtained using PCR-RAPD, with the help of some analytical software. The method yielded high quality and quantity of DNA. Six random primers (OPA-3, OPA-4, OPA-13, OPA-18, OPC-5 and OPC-6) were found to give good amplifications of *E. longifolia* DNA samples [23]. Some scientists are interested in the *in vitro* production of the *E. longifolia* plantlets or plant tissues for sustainable production of active ingredients [24,25,26,27,28,29,30,31]. Ling *et al.* developed a protocol to optimize protoplast isolation from callus of *E. longifolia* [32]. Most recently, Lulu *et al.*, optimized the conditions for the production of adventitious roots from *E. longifolia*, in balloon-type bubble bioreactor cultures, suitable for the large-scale commercial production of its roots containing high yield of bioactive compounds [33].

## 2. Historical or Traditional Uses

*E. longifolia* is used to cure lumbago and indigestion. It is used as a power tonic after delivery, and use for treatment of fever, jaundice, cachexia, and dropsy [12,34]. *E. longifolia* is one of the most popular folk medicines for its aphrodisiac effects and treatment of intermittent fever (malaria) [35]. Decoctions of *E. longifolia* leaves are used for washing itches, while its fruits are used in curing dysentery [12]. Its bark is mostly used as a vermifuge [12], while the taproots are used to treat high blood pressure, and the root bark is used for the treatment of diarrhea and fever [36]. Mostly the roots extract of *E. longifolia* are used as folk medicine for sexual dysfunction, aging, malaria, cancer, diabetes, anxiety, aches, constipation, exercise recovery, fever, increased energy, increased strength, leukemia, osteoporosis, stress, syphilis and glandular swelling, as well as it is used as an aphrodisiac, antibiotic, appetite stimulant and health supplement [36,37,38,39,40,41,42].

Traditionally, the water decoction of *E. longifolia* root is consumed. Nowadays, more convenient formulas are available, primarily additives mixed with teas and coffees, and over 200 products are available either in the form of raw crude root powder or as capsules mixed with other herbs in the health-food market [7]. Due to the many traditional and scientific benefits, there has been a demand for *E. longifolia* products with over 200 *E. longifolia* products registered with the National Pharmaceutical Control Bureau of Malaysia (NPCB, 2016). It is now currently sold as a Traditional Herbal Medicine in Malaysia. Approximately 21,000 kg of *E.* *longifolia* are harvested by collectors per year, with a demand of approximately >54,000 kg per year.

## 3. Chemical Constituents

The wide spectrum of pharmacological effects was closely associated with various biologically active compounds of *E. longifolia* roots, stem, leaves and even bark. Kuo *et al.*, reported the isolation of sixty five phenolic compounds from the *E. longifolia* root [36]. *E. longifolia* is a rich source of various classes of bioactive compounds, which includes quassinoids, β-carboline alkaloids, canthin-6-one alkaloids, triterpene-type tirucallane, squalene derivatives, and eurycolactone, eurycomalactone, laurycolactone, biphenyl neolignan and bioactive steroids [7,36,42,43,44,45]. Among these, bitter tasting quassinoid phytoconstituents account for a major portion in the *E*. *longifolia* root contents. The quassinoids are a group of nortriterpenoids with dynamic pharmacological properties [40]. Quassinoids, are even effective at inhibiting cell growth in nanomolar and subnanomolar concentrations [41]. The presence of tirucallane and squalene-type triterpenes might be the quassinoids’ biological precursors. β-Carboline and Canthin-6-one alkaloids formed as metabolic by-products are natural amine compounds that repel herbivores and insects [46]. The metabolite type and concentration in *E*. *longifolia* plant extracts, depend on the processing temperature as well as geographical factors. For standardization, it is crucial to ensure the consistency of the chemical bioactive components, particularly for the efficacy of herbal medicines [47]. Summarized here are some major constituents of *E*. *longifolia* with their secondary metabolites:

Quassinoids, including various types of eurycomanone (pasakbumin-A), eurycomanols, pasakbumin-B, hydroxyklaineanones, eurycomalactones, eurycomadilactones, eurylactones, laurycolactones, longilactones, and hydroxyglaucarubol have been isolated from the roots of *E. longifolia* [38,39,48,49,50,51,52].

The squalene derivatives include teurilene, eurylene; 14-deacetyleurylene; and longilene peroxide [53,54].

The biphenyl neolignans class includes; 2-hydroxy-3,2,6-trimethoxy-4-(2,3-epoxy-1-hydroxypropyl)-5-(3-hydroxy-1-propenyl)-biphenyl; two isomeric 2,2-dimethoxy-4-(3-hydroxy-1-propenyl)-4-(1,2,3-trihydroxypropyl) diphenyl ethers; and 2-hydroxy-3,2-dimethoxy-4-(2,3-epoxy-1-hydroxypropyl)-5-(3-hydroxy-1-propenyl)biphenyl [55].

Alkaloids included 5,9-dimethoxycanthin-6-one; 9,10-dimethoxycanthin-6-one, 11-hydroxy-10-methoxycanthin-6-one; 10-hydroxy-9-methoxycanthin-6-one; and 9-methoxy-3-methylcanthin-5,6-dione [45,56,57].

**Figure 1:**
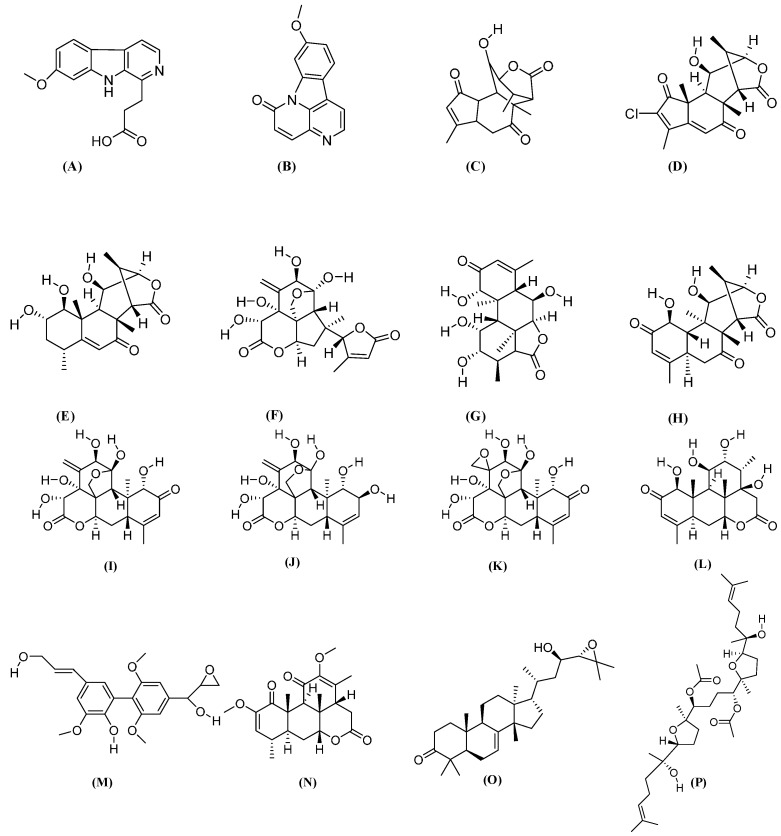
Chemical structures of various biological active constituents from E. longifolia; (**A**) 7-Methoxy-beta-carboline-1-propionic acid (C15); (**B**) 9-methoxycanthin-6-one (C15); (**C**) Laurycolactone (C17); (**D**) Eurycolactone B (C18); (**E**) Eurycomalide A (C19); (**F**) Eurylactone (C19); (**G**) Longilactone (C19); (**H**) Eurycomalactone (C19); (**I**) Eurycomanone (C20); (**J**) Eurycomanol (C20); (**K**) Pasakbumin B (C20); (**L**) Hydroxyklaineanone (C20); (**M**) Biphenyl-neolignan (C21); (**N**) Quassin (C22, basic ring of quassinoids); (**O**) Niloticin (C30); and (**P**) Eurylene (C34).

Major isolated chemical constituents with metabolites from *E. longifolia* Jack and their pharmacological effects, are listed in Table 1, while their chemical structures are presented in Figure 1.

## 4. Analytical Methods

Besides the major constituents, secondary metabolites are usually present in a small amount. That’s why, high sensitivity and high mass accuracy is required to produce reliable data. Mostly, data from IR, UV, MS and X-ray analysis was evaluated further for ^1^H- and ^13^C-NMR spectral analysis. These procedures for identification of unknown entity, require high purity as well as high concentration of extracted compounds.

Today, mass spectrometry is the most specific and versatile method of detection in liquid chromatography, especially perfect for the analysis of some multiple components pharmaceutical and herbal products [93,94]. Liquid chromatography with mass spectrometry (LC-MS) is recognized as a most suitable and powerful tool for identification as well as quantification of various herbal product and their constituents [95,96,97]. From the plant kingdom, quassinoids are bitter constituents found exclusively in various species of the Simarouboidaea (a subfamily of the Simaroubaceae) and are biogenetically degraded triterpenes displaying a wide range of physiological properties *in vitro* and/or *in vivo* [98,99]. Numerous research reports are available on liquid chromatography methods for the analysis of quassinoid *E. longifolia* bio-constituents, using photodiode array or fluorescence and UV detection. However, none of these methods are sensitive enough to detect nonchromophoric bioactive constituents, such as eurycomanol present in *E. longifolia* [57,58,100], so mass spectrometry is the best option for analysis of all constituents and secondary metabolites from *E. longifolia*. Chua *et al.*, used a number of three liquid chromatography mass spectrometry hybrid systems (QTof, QTrap and TripleTof), to scan for small metabolites and also to detect the targeted metabolites, such as alkaloids, quassinoids, triterpene and biphenylneolignans from *E. longifolia* extracts [47]. Teh *et al.*, developed and optimized a LC-MS method using ESI in a positive ion mode for bioactive compounds simultaneous determination, from *E. longifolia* [101]. Recently, liquid chromatography-tandem mass spectrometry method for the simultaneous determination of six major quassinoids of *E. longifolia* *i.e.*, eurycomanone, 13α(21)-epoxyeurycomanone, 13,21-dihydroeurycomanone, 14,15β-dihydroxy-klaineanone, longilactone and eurycomalactone was developed. By using a LC-MS method, the content of these quassinoids was measured in in dietary supplement tablets and capsules, to confirm the purity of *E. longifolia* in commercial products [102]. For quick screening of sildenafil analogues in *E. longifolia* products, a two-tier screening method using a near infrared (NIR) spectral database was developed. This method has allowed rapid screening on the test samples to verify their content as labelled despite not having the spectra of those products in the database. It could be used for product identification, drug screening for mixed adulteration as well as drug quality surveillance, particularly in cases where reference samples are difficult to obtain [103].

## 5. Evidenced-Based Pharmacology

### 5.1. Male Fertility Enhancement Effect

Infertility is a major clinical problem, which affects the people medically, economically and psychosocially. Almost, 15% of all couples in the U.S. are infertile, and it is predicted that the male factor is responsible in many of such cases [104]. Male infertility refers to a male’s inability to achieve a pregnancy in a fertile female. In humans, this accounts for 40%–50% of infertility cases [105,106]. Infertility in males is a multifactorial disease, based on numerous factors including reduced spermatogenesis and also production of dysfunctional sperm, which are the major prevalent underlying characteristic in idiopathic male infertility cases [107,108]. One meta-analysis of sixty-one studies worldwide reported s downward trend in the sperm count and semen volume over the past fifty years [109,110].

Mostly, the water-soluble *E. longifolia* extracts were reported to be able to enhance male fertility (with regards to higher semen volumes, spermatozoa count, and motility) in rodents [111,112] and in human trials [86,113,114].

The standardized extract F2 of *E. longifolia* (25mg/kg *p.o*) and its major quassinoids, especially eurycomanone (250 mg/kg *p.o*) improved rat spermatogenesis by affecting the hypothalamic-pituitary-gonadal axis and the potential efficacy may be worthy of further investigation [111].

Eurycomanone, the major quassinoid in the *E. longifolia* root extract, significantly increased testosterone production on a dose-dependent manner at 0.1, 1.0 and 10.0 μM (*p* < 0.05). It enhanced testosterone steroidogenesis at the rat testicular Leydig’s cells by inhibiting aromatase conversion of testosterone to oestrogen, and may also involve in phosphodiesterase inhibition at a high concentration, so authors have suggested that quassinoids from *E. longifolia* may be worthy for further development as new phytomedicines for the treatment of testosterone-deficient idiopathic male infertility and sterility [112]. Also, standardized extracts of *E. longifolia* Jack containing a high concentration of quassinoids (20% eurycomanone and 4% of 13α,21-dihydroeurycomanone) may have potential anti-estrogenic effects [86].

The quassinoid-containing *E. longifolia* extract affects male infertility by suppressing α-2-HS glycoprotein expression, which indirectly increases the testosterone levels and insulin sensitivity. They indicated that serum α-2-HS glycoprotein was reduced in rats treated with standardized *E. longifolia* extract, which will provide rational for further investigation in animal models of infertility with diabetes [113].

A randomized, double-blind, placebo-controlled, parallel group study was conducted to investigate the aphrodisiac clinical evidence of *E. longifolia* extract in men. The total twelve weeks’ study in 109-men between 30- and 55- years of age, divided in a group of 300 mg of water extract of *E. longifoli*a (Physta^®^)-treated and placebo. The *E. longifoli*a group showed higher scores in the overall erectile-function-domain (IIEF, *p* < 0.001), the sexual libido (14% by week 12), Seminal Fluid Analysis (SFA)-with sperm motility at 44.4%, and semen-volume at 18.2% after treatment [114].

Chan *et al.*, statistically analyzed the spermatozoa count, morphology, motility, plasma testosterone level and Leydig cell count of the animals by ANOVA. Their results showed that the sperm counts of rats given the standardized methanol extract alone at doses of 50, 100 and 200 mg/kg were increased by 78.9%, 94.3% and 99.2%, respectively, when compared with that of control (*p* < 0.01) [115].

Ang and Ngai showed that the fractions of *E. longifolia* Jack (0.5 g/kg) decreased the hesitation time. Furthermore, they caused a transient increase in the percentage of the male rats responding to the right choice; more than 50% of the male rats scored “right choice”; using the electrical copulation cage [116].

*E. longifolia* has been shown to elevate serum testosterone and increased muscle strength in humans. Chen *et al.*, investigated the effects of standardized water extract of *E. longifolia* (Physta^®^) at a dose of 400 mg/day for 6 weeks on testosterone: epitestosterone (T:E) ratio, liver and renal functions in male recreational athletes found no significant difference between the results of supplementation results and placebo [117].

Study on the sexual qualities of middle-aged male rats after dosing with 0.5 g/kg of various fractions of *E. longifolia*, showed that it enhanced the sexual qualities of the middle-aged male rats by decreasing their hesitation time as compared to controls [118].

A randomized, double-blind, study with placebo-controlled was conducted for proprietary freeze-dried water extract of *E. longifolia* (Physta^®^) effects on sexual performance and well-being in men. For this study, men aged 40–65 years were screened for 12-week. Results showed the significant improvements in scores for the sexual intercourse attempt diary, erection hardness scale, sexual health inventory of men, and aging male symptom scale (*p* < 0.05 for all), concluded that Physta^®^ was well tolerated and more effective than placebo in enhancing sexual performance in healthy volunteers [59].

*E. longifolia* extract acts as a potential agent to increase spermatogenesis and sperm counts, and for reversing the effects of estrogen in rats, after fourteen consecutive days of treatment [119].

In other study, Ang *et al.*, showed that *E. longifolia* produced a dose-dependent, recurrent and significant increase in the episodes of penile reflexes as evidenced by increases in quick flips, long flips and erections of the treated male rats during 30 min observation period [17].

According to Tambi and Imran’s investigations, 350 patients were given 200 mg of the *E. longifolia* extract daily, and follow-up semen analyses were performed every 3 months up to 9 months. These patients showed significant improvement in all semen parameters, allowing for 11 (14.7%) spontaneous pregnancies [120].

Erasmus *et al.*, treated semen samples with *E. longifolia* extract (*in vitro* condition), found a significant dose-dependent trends for vitality, total motility, acrosome reaction and reactive oxygen species-positive spermatozoa; but no deleterious effects on sperm functions at therapeutically used concentrations (<2.5 µg mL^−1^) [121].

An increase in sperm count, motility and viability in rats, when treated with aqueous *E. longifolia* extract. Noor *et al.*, investigated that *E. longifolia* can increase sexual behavior of male rats and the sperm quality; which were found to be dose dependent [122]. One study indicates that *E. longifolia* exerts proandrogenic effects that enhance the testosterone level [123].

The *in vivo* effect of aqueous extract of *E. longifolia* was investigated on body and organ weight as well as functional sperm parameters in terms of safety and efficacy in the management of male infertility, in male rats. Testosterone concentration increased by 30.2%, total sperm concentration, progressive motility and vitality significantly increased, MMP improved markedly by 25.1%, with increased in muscle weight, non-significantly, so it appears that *E. longifolia* use is safe for possible treatment of male infertility and ageing male problems [124].

In human studies, Tambi *et al.*, treated a group of patients suffering from late-onset hypogonadism (LOH) with Tongkat ali extract, which showed significantly (*p* < 0.0001) improved the Ageing Males’ Symptoms (AMS) score as well as the serum testosterone concentration. Thus, Tongkat ali extract appears to be useful as a supplement in overcoming the symptoms of LOH and for the management of hypogonadism [125].

The testosterone deficiency syndrome (TDS), can be characterised by numerous symptoms, including low libido, fatigue, increased fat mass, osteoporosis or erectile dysfunction, and up-to 80% of men have experience some sort of ageing male symptoms. Conventionally, TDS is treated with testosterone replacement therapy (TRT). With the beneficial effects of this therapy, significant adverse effects have been indicated, including prostate cancer. *E. longifolia* is the herbal alternative to TRT, which has been shown to successfully restore serum testosterone levels, and significantly improve the physical condition and sexual health of patients. Therefore, *E. longifolia* might be considered a safe alternative to TRT [126].

For the copulatory activity of sexually sluggish rats, with acute (500, and 1000 mg/kg) and also subacute treatments with *E. longifolia* root powder, significantly reduced ejaculation latencies, and increased the percentage of mounting and ejaculating animals; while the subacute administration reduced post-ejaculatory interval. In case of impotent rats, both treatments increased the percentage of mounting and ejaculating rats. Serum testosterone levels were increased in rats that were treated subacutely, in comparison with control [127].

One experiment by Ang and Sim showed that *E. Iongifolia* Jack continued to enhance and also maintain a high level of both the total number of successful crossovers, mountings, intromissions and ejaculations during the 9–12th week observation period [128].

In animal research, an herbal combination containing *Panax quinquefolius*, *Eurycoma longifolia*, *Epimedium grandiflorum*, *Centella asiatica*, and flower pollen extracts enhanced erectile function [129]. Improvements were noted in the penile erection index (PEI). In boars, an herbal preparation containing *Eurycoma longifolia*, *Tribulus terrestris*, and *Leuzea carthamoides* increased libido (by 20%) and semen quality (volume, concentration, *etc.*) [130].

Randomized controlled trials investigating *E. longifolia* compared to placebo were included by Kotirum *et al.* and suggests that *E. longifolia* root extract may have a clinical benefit on improving erectile dysfunction performance. Based on current evidence, the herbal extract of *E. longifolia* may have clinical effect on erectile function, but needs further clinical evidence of efficacy trials to make any firm recommendation [131].

In a pilot study, Henkel *et al.* investigated the ergogenic effect of *E. longifolia* in elderly people and found that it is a potential herbal supplement for physically active aged male and female (age 57–72 years). Treatment resulted in significant increases in total and free testosterone concentrations and muscular force in men and women, when *E. longifolia* extract 400 mg/day was used for 5 weeks [132].

### 5.2. Antimalarial Effect

The WHO estimates that in 2013, there were 207 million annual cases of malaria, resulting in 627,000 deaths, from *Plasmodium falciparum* [133,134]. There are about 10,000 malaria cases per year in Western Europe, and 1300–1500 in the United States [135]. *E. longifolia* extract is traditionally used for malarial fevers and has good anti-malarial effect against *P. falciparum.*

Chan *et al.*, tested the extracts of *E. longifolia* for antiplasmodial activity against a multi-drug resistant Thailand’s strain (K-1) of *P. falciparum* under *in vitro* conditions. They isolated 10-hydroxycanthin-6-one, eurycomalactone, eurycomanone and eurycomanol from the plant, which showed antimalarial activities [60].

According to Kardono *et al.*, two compounds, eurycomanone and 7-methoxy-β-carboline-1-propionic acid showed significant antimalarial activity against *P. falciparum* strains [61]. Low *et al.*, concluded that the administration of the bioactive standardized extract Fr2 (200 mg/kg) showed a good antimalarial effect. 13α(21)-epoxyeurycomanone and eurycomanone may be the only quassinoids contributing to the overall antimalarial activity of *E. longifolia* [62].

In study, conducted during 2008 in Mae Sot, Tailand, a standardized extract of *E. longifolia* containing three major quassinoids, eurycomanone (**1**), 13,21-dihydroeurycomanone (**2**) and 13α(21)-epoxyeurycomanone (**3**) was evaluated for antiplasmodial activity against *Plasmodium falciparum*. Activity was compared with that of artemisinin, using thirty-eight fresh parasite isolates and assessment of inhibition of schizont maturation. The IC_50_, IC_90_ and IC_99_ values for artemisinin were 4.30, 45.48 and 310.97 μg/L, and those for the root extract from *E. longifolia* 14.72, 139.65 and 874.15 μg/L respectively. The inhibitory activity of the *E. longifolia* extract was higher than that expected from the three quassinoids isolated from the plant, suggesting synergism between the quassinoids or the presence of other unidentified compounds [63].

Ang *et al.*, tested *E longifolia* extract activity *in vitro* on Malaysian chloroquine-resistant Plasmodium falciparum culture. They showed that the antimalarial activity of *E. longifolia* Jack was dose-dependent and reached a maximum of <50% at 0.07−5.00 μg·mL^−1^ after 1-day post-treatment. However, complete inhibitions were observed at 1.25–5.00 μg·mL^−1^ extract after 3 days’ post-treatment and 0.62 and 0.31 μg·mL^−1^ after 4 and 6 days’ post-treatment, respectively [64].

*E. longifolia* methanol extract (TA164) decreased the glutathione (GSH) content of both infected and healthy erythrocytes at a certain dosage and incubation period. Both effects of TA164 to GSH content of host or parasite can be the cause of *P. falciparum* growth inhibition *in vitro* and screening the activity of GSH synthesis can be one of the procedures in evaluating the antimalarial properties of herbal products [136].

### 5.3. Cytotoxic and Anti-Proliferative Effect

Cytotoxic effects of novel drug entities and traditional medicines are very essential to be investigated before testing their further pharmacological activity. After establishment of positive cytotoxic effects, anti-proliferative effects (rate of cytotoxicity) are also investigated to check and confirm their further anti-cancer effectiveness, using *in vitro* as well as *in vivo* models. Various constituents from *E. longifolia* have been tested for cytotoxic effects, and some of these also showed positive anti-proliferative effects.

Cancer, medically known as a malignant neoplasm, is a broad group of diseases involving unregulated cells. In malignant neoplasm (cancer), cells divide and grow uncontrollably, forming malignant tumors, and invading nearby parts of the body. It may also spread to more distant parts of the body through the lymphatic system or bloodstream. Over 200 different known cancers that can affect humans; and there are over sixty different organs in the body where a cancer can develop. A statistical report in 2012 showed that total 338,623 people were diagnosed with cancer in the UK, while 161,823 deaths from cancer ocurred (survival rate was 50%) [137].

*E. longifolia* has cytotoxicity and antiproliferative effects on various human cancer cell lines, as well as various solid tumors, including lung, breast and cervical cancers. Kuo *et al.*, [36] isolated and identified nearly 65 compounds from the roots of *E. longifolia* and screened them for the potential cytotoxicity and anti-HIV activities by *in vitro* assays. Among the compounds evaluated, 13β,21-dihydroxyeurycomanol [60], 6-dehydroxylongilactone [72], 9-methoxycanthin-6-one [75], canthin-6-one [76], eurylene [53], 9-hydroxycanthin-6-one [76], longilactone [75], 9-methoxycanthin-6-one 3*N*-oxide [76], 14,15β-dihydroxyklaineanone [75], pasakbumin C [50], canthin-6-one 9-*O*-β-glucopyranoside [76], were screened for *in vitro* cytotoxicity against A-549 and MCF-7 tumor cell lines [138] and no inhibition of HIV replication in H9 lymphocytes except for eurylene and pasakbumin B [139]. Compounds longilactone, 6-dehydroxylongilactone, 9-methoxycanthin-6-one, canthin-6-one, longilactone, 9-methoxycanthin-6-one, 14,15β-dihydroxyklaineanone, pasakbumin C, and canthin-6-one 9-*O*-β-glucopyranoside demonstrated strong cytotoxicity toward A-549 cell lines, however, longilactone, 6-dehydroxylongilactone, 9-methoxycanthin-6-one, eurycomanone, pasakbumin B, and 9-methoxycanthin-6-one displayed strong cytoxicity toward the MCF-7 cell line.

According to Park *et al.*, [51] the compounds eurycomalactone [49], longilactone [140], and 14,15β-dihydroxyklaineanone [140] showed significant cytotoxicity in both A549 and MCF-7, while 13,21-dihydroeurycomanone [140] was more selective against A549 and eurycomanone [140] showed cytotoxic effects only against MCF-7. In the HeLa cell line, compounds eurycomalactone, 13,21-dihydroeurycomanone, eurycomanone, 13α(21)-epoxyeurycomanone, longilactone, and 14,15β-dihydroxyklaineanone displayed significant cytotoxicity showing the relative cell viability ranging from 21.01% ± 2.46% to 66.9% ± 6.67% at the concentration of 100 μM.

Three new [*n*-pentyl β-carboline-1-propionate, 5-hydroxymethyl-9-methoxycanthin-6-one, and 1-hydroxy-9-methoxycanthin-6-one] and 19 known β-carboline alkaloids were isolated from the roots of *E. longifolia*. These compounds were screened for *in vitro* cytotoxic activities; in which 9-methoxycanthin-6-one and canthin-6-one demonstrated significant cytotoxicity against human lung cancer (A-549) and human breast cancer (MCF-7) cell lines [76].

Kardono *et al.*, isolated and characterized five cytotoxic constituents from the roots of *E. longifolia*. Four of the canthin-6-one alkaloids, namely, 9-methoxycanthin-6-one, 9-methoxycanthin-6-one-*N*-oxide, 9-hydroxycanthin-6-one, and 9-hydroxycanthin-6-one-*N*-oxide and one quassinoid, eurycomanone, were found to possess cytotoxic effects against a panel of cell lines like: human cancer cell types (breast, colon, fibrosarcoma, lung, melanoma, KB, and KB-V1) and murine lymphocytic leukemia (P-388) [61].

Eurycomanone is a cytotoxic bioactive ingredient found in *E. longifolia* Jack, that has a cytotoxic response against many epithelial cell types. The antiproliferative activity of eurycomanone was investigated on cancerous cell lines (Caov-3, HeLa, Hep G2, HM3KO and MCF-7) and it was found to be relatively nontoxic on noncancerous cell lines (MDBK, Vero). Eurycomanone proved to be cytotoxic towards HeLa cells by triggering apoptotic cell death [141].

Tong *et al.* investigated the *in vitro* and *in vivo* anti-cancer activities of a standardized quassinoid mixture (SQ40) from *E. longifolia* on LNCaP human prostate cancer cells, and showed that it induced selective cytotoxicity on human prostate cancer cells and inhibited the growth of LNCaP cells. SQ40 down-regulated the expression levels of G_1_-to-S phase transition regulatory proteins, cyclin D1, CDK4 and CDK2 and up-regulated cyclin inhibitor protein, p21^Waf1/Cip1^ which subsequently led to cell cycle arrest in G_0_/G_1_ phase. The anti-tumorigenic activity of SQ40 was successfully demonstrated in the mouse xenograft model [142].

Recently, Hajjouli *et al.* concluded that *E. longifolia* constituents, eurycomanone and eurycomanol are the regulators of signaling pathways involved in proliferation, cell death and inflammation. Both eurycomanone and eurycomanol inhibited Jurkat and K562 cell viability and proliferation without affecting healthy cells. Furthermore, eurycomanone inhibited NF-κB signaling pathway through inhibition of IκBα phosphorylation and upstream MAPK (mitogen activated protein kinase) signaling. Eurycomanone and eurycomanol present differential toxicity towards leukemia cells, and eurycomanone having the α,β-unsaturated ketone could be prerequisite for the NF-κB inhibition [143].

Wnt signaling regulates various processes such as cell proliferation, differentiation, and embryo development. 9-hydroxycanthin-6-one, decreased the expression of Wnt signal target genes, mitf and zic2a, through the activation of GSK3β independent of CK1α [144].

The quassinoids isolated from *E. longifolia* have been studied for thir *in vitro* cytotoxicities against KB cells derived from human epidermoid carcinoma of the nasopharynx [140]. Itokawa *et al.*, isolated a new squalene-type triterpene, named eurylene, from *E. longifolia* which were found to be cytotoxic [53]. Chan *et al.*, isolated a new C19 quassinoid 6α-hydroxyeurycomalactone from the roots of *E. longifolia* and have reported that the cytotoxic activity of these quassinoids was not mediated through DNA cleaving properties [49].

Chronic myelocytic leukemia (CML) is a malignant disease of the human hematopoietic stem cell which is characterized by a marked increase in granulocytes bone marrow hyperplasia and splenomegaly [145]. CML accounts for 15–20 percent of all leukemias [145,146] with a worldwide incidence of 1–2/100,000 [147,148,149]. The various isolates and purified eurycomane, an active compound from the roots of *E. longifolia*, were examined for their cytotoxic effect in K-562 cells isolated from patients with chronic myelocytic leukaemia (CML).

Al-Salahi *et al.*, assessed the *in vitro* and *in vivo* anti-proliferative and apoptotic potentials of *E. longifolia* on K-562 leukemic cell line. Intraperitoneal administration of TAF273 (*E. longifolia* fraction, 50 mg/kg) resulted in a significant growth inhibition of subcutaneous tumor. TAF273 showed potent anti-proliferative activity *in vitro* and *in vivo* models of Chronic Myelogenous Leukemia (CML) and therefore, justifies further efforts to define more clearly the potential benefits of using TAF273 as a novel therapeutic strategy for CML management [150]. The cytotoxic activity of quassinoids was not found to be mediated through DNA cleaving properties [49]. *In vitro*, the anticancer effects of a fraction of *E. longifolia* were due to apoptosis via a caspase-9 and p53-independent manner [151] that perhaps involved Bcl-2 protein [152].

Angiogenesis, a process of formation of new branches of blood vessels, is strongly implicated in several important physiological situations [153,154]. Dysregulation of angiogenesis is involved in several pathological conditions, including atherosclerosis, proliferative retinopathies, rheumatoid arthritis, psoriasis, tumor growth and metastasis [155]. It is well recognized that angiogenesis is essential for the growth and metastasis of most solid malignancies, an increased body of evidence supports the enhancement of angiogenesis in hematologic malignancies as well [156]. Therefore, angiogenesis is currently becoming an important target for chemotherapeutic approaches in cancer therapy [157].

Antiangiogenic potential of partially purified quassinoid-rich fraction (TAF273) of *E. longifolia* root extract was evaluated using *ex vivo* and *in vivo* angiogenesis models and the anti-angiogenic efficacy of TAF273 were investigated in human umbilical vein endothelial cells (HUVEC). *In vivo*, it causes significant suppression in sprouting of microvessels in the rat aorta (IC_50_, 11.5 μg/mL), and shows a remarkable inhibition (63.13%) of neovascularization in chorioallantoic membrane of the chick embryo (IC_50_, 50 μg/mL). *In vitro*, TAF273 significantly inhibited the major angiogenesis steps such as proliferation, migration and differentiation of HUVECs. Thus, *E*. *longifolia* could be the potential source of promising therapeutic agents to treat angiogenesis-related disorders [158].

Fractions of *E. longifolia* extract have also been reported to induce apoptosis in breast cancer cells [152]. Further, Tee *et al.*, elucidated the mode of action of F16 (a plant-derived pharmacologically active fraction) and observed that the intrinsic apoptotic pathway was invoked, with the reduction of Bcl-2 protein. It was concluded that the F16 from *E. longifolia* exerts anti-proliferative action and growth inhibition on MCF-7 cells through apoptosis induction, and that it may have anticancer properties [151].

The anti-proliferative, apoptotic and differentiating activities of partially purified sub-fractions (F1–F3) of *E. longifolia* root extracts were investigated on HL-60 leukemic cells. F1 showed unremarkable growth inhibition rate while F2 and F3 showed growth inhibitory effects with median inhibitory concentration (IC_50_) values of 15.2 and 28.6 µg/mL, respectively. *E. longifolia* extract (F2) showed promising anti-leukemic activity and can be a candidate for the development of a drug for the treatment of acute promyelocytic leukemia (APL) [159].

Nurhanan *et al.*, evaluated the methanol, *n*-butanol, chloroform and water extracts obtained from the root of *E. longifolia* for its possible cytotoxic effect against KB, DU-145, RD, MCF-7, CaOV-3, and MDBK cell lines. Their results indicated that except for the water extract, all the other extracts produced significant cytotoxic effecte on these cell lines with no significant cytotoxic effect on MDBK (kidney) normal cell line. An alkaloid, 9-methoxycanthin-6-one was detected in each extract with different intensities, and was envisaged to be responsible for the observed activities [160].

Razak *et al.*, reported that the extract of *E. longifolia* is found to be cytotoxic with IC_50_ of 11 μg/mL and 13 μg/mL on Hep2 and HFL1 cell lines respectively and that the combined extracts of *E. longifolia* and *Hunteria zeylanica* are more cytotoxic than the single extract on Hep2 cell lines [161].

### 5.4. Antimicrobial Effects

Farouk *et al.*, showed that the alcoholic and acetone extracts of the leaves and stem were active on both the Gram-positive and Gram-negative bacteria *Escherichia coli* and *Salmonella typhi*. The root extracts had no antibacterial activity against the Gram-positive and Gram-negative bacteria tested. Aqueous leaves extract showed antibacterial activity against *Staphylococcus aureus* and *Serratia marscesens* [162].

Extracts from *E. longifolia* and *L. pumila* leaves were evaluated and analyzed for their antibacterial activity against human pathogenic Gram positive (*Staphylococcus aureus*) and Gram negative (*Pseudomonas aeruginosa*) bacteria. The extracts were prepared in different solvents (acetone, methanol, ethanol, and phosphate buffer) and at various concentrations ranging from 5 to 100 mg/mL. Most of the extracts showed relatively high antibacterial activity against the tested bacteria with inhibition zone diameters ranging between 7 and 25 mm. The minimum concentration of *E. longifolia* and *L. pumila* extracts which inhibited the growth of *S. aureus* and *P. aeruginosa* was 75 mg/mL in ethanol and 25 mg/mL in a phosphate buffer, respectively [163].

Kong *et al.* screened natural extracts from six plants, including *E. longifolia*, that improved the survival of *S. aureus*-infected worms by at least 2.8-fold, suggesting that these extracts could possibly activate host immunity to eliminate the bacteria or possibly interfere with the factor/s that prevent pathogen accumulation [164].

### 5.5. Anti-Inflammatory Effects

It was demonstrated that the β-carboline alkaloid 7-MCPA (7-methoxy-(9*H*-β-carbolin-1-yl)-(*E*)-1-propenoic acid) isolated from *E. longifolia* hairy-root cultures activated Nrf2 via a ROS-dependent p38 MAPK pathway and 7-MCPA anti-inflammatory effects was associated with 7-MCPA-induced activation of the Nrf2/HO-1 pathway. This study clarified the molecular mechanisms underlying the anti-inflammatory activities of β-carboline alkaloids of *E. longifolia*, which may be useful to prevent or treat inflammatory diseases [165].

Eurycomalactone, 14,15β-dihydroklaieanone, and 13,21-dehydroeurycomanone were identified as potent NF-κB inhibitors with IC50 values of <1 μM [45]. Varghese *et al*, studied hydroalcoholic extract of *E. longifolia* Jack for its antioxidant and *in vitro* anti-inflammatory properties. The antioxidant activity (free radical scavenging) was evaluated to determine the total antioxidant capacity of extract *E. longifolia*. The DPHH assay showed significant antioxidant activity in all concentrations used (*i.e.,* 10, 25, 50, 100 and 250 µg/mL). The human RBC (HRBC) stabilization method was utilized to evaluate the *in vitro* anti-inflammatory activity of the extract, and it was found that this anti-inflammatory activity increased in a concentration dependent manner [82].

### 5.6. Anti-Anxiolytic Effect

The anti-anxiety effect of various fractions of *E. longifolia* was investigated in mice using various behavioral tests, including the open field (emotional state), elevated plus-maze (anxiolytic and anxiogenic drug effects), and anti-fighting test. The *E. longifolia* anxiolytic effect was similar to that of the positive control diazepam [166].

In human, effects of *E. longifolia* hot-water extract was screened for stress hormones and mood state in 63 subjects (32 men and 31 women) for moderate stress, with placebo for 4 weeks, and indicates that daily supplementation with *E. longifolia* extract improves stress hormone profile and certain mood state parameters [167].

### 5.7. Antidiabetic Effect

Blood glucose decreased in streptozotocin-induced hyperglycemic adult rats after treatment of 150 mg/kg body weight using aqueous extracts of *E. longifolia*. Blood-glucose levels decreased 38% (*p* < 0.05) and 47% (*p* < 0.001) for two different *E. longifolia* extracts. In normoglycaemic rats, no significant reduction was noted when the same extracts were used [168].

*E*. *longifolia* root extract increased insulin sensitivity through the enhancement of glucose uptake by more than 200% at 50 μg/mL and suppressed lipid accumulation in a concentration-dependent manner, suggesting the ability of *E. longifolia* to suppress lipid production would provide additional benefits in the treatment of diabetes [169].

### 5.8. Osteoporosis Preventive Effect

Osteoporosis in men is attracting more interest as it is becoming one of the main causes of morbidity and mortality in older men. Approximately 2 million men in the United States suffer from osteoporosis [170]. Worldwide, 1 in 3 women over 50 will experience osteoporotic fractures, as will 1 in 5 men [171,172,173]. According to Tambi and Kamarul, *E. longifolia* contains high concentrations of superoxide dismutase (SOD), an antioxidant that plays an important role in counteracting oxidative stress [120]. Other components of *E. longifolia*, such as alkaloids and triterpenes, can also act as antioxidants that may reduce bone loss and maintain the bone formation rate [123].

Recently, it was established that *E. longifolia* may be used in the prevention and treatment of osteoporosis, or more specifically, male osteoporosis. Shuid *et al.*, showed that both testosterone replacement and *E. longifolia* supplementation to orchidectomised rats were able to maintain the bone calcium levels, with the former showing better effects, so *E. longifolia* prevented bone calcium loss in orchidectomised rats and therefore, has the potential to be used as an alternative treatment for androgen deficient osteoporosis [174]. The bioactive complex polypeptides from the *E. longifolia* root extract, labelled as eurypeptides, can exert and enhance their effects on the biosynthesis of various androgens [175]. Eurypeptides work by stimulating dihydroepiandosterone (DHEA). DHEA in turn will act on androgen receptors to initiate the conversion of androstenedione and androstenediol to testosterone and estrogen, respectively [125]. These eurypeptides may also alleviate SHBG and subsequently increase the free testosterone level [176]. Due to these proandrogen properties of *E. longifolia*, it is able to stimulate osteoblast proliferation and differentiation, resulting in increased bone formation rate. High levels of testosterone and estrogen may also exert proapoptotic effects on osteoclasts, reducing the bone resorptive activity. As testosterone levels decrease with age, it has been suggested that men can consume *E. longifolia* (at suitable dosages) as a supplement [177]. Other than its proandrogenic properties, *E. longifolia* contains high levels of nitric oxide (NO) [178] that have effects on bone.

Male osteoporosis can also be explained in terms of an oxidative stress mechanism. Free radicals, mainly reactive oxygen species (ROS), are efficiently scavenged in the body. However, oxidative stress will occur when there is an imbalance between increased ROS levels and inadequate antioxidant activity [179]. Orchidectomy (a model of androgen-deficient osteoporosis), can promote up-regulation of ROS which leads to oxidative stress. Oxidative stress plays a role in osteoblast apoptosis and osteoclast differentiation [180]. There are several mechanisms proposed for its antiosteoporotic effects. The main mechanism is via its testosterone-enhancing effects for the prevention and treatment of androgen-deficient osteoporosis. Other mechanisms involved are through its nitric oxide generation and antioxidative properties. Due to *E. longifolia*’s safety profile and potential as an alternative antiosteoporotic agent, further studies are warranted to document a better and conclusive mechanism for its therapeutic action [123].

Androgen-deficient osteoporosis in men is treated with testosterone therapy, which is associated with many side effects. *E. longifolia* is known to possess androgenic properties and has been reported to protect bone from androgen-deficient osteoporosis in experimental animal models [181]. The combination therapy of *E. longifolia* and low-dose testosterone has potential for treatment of androgen-deficient osteoporosis. The lower testosterone dose is beneficial in reducing the side effects of testosterone therapy [181]. *E. longifolia* exerts proandrogenic effects that enhance testosterone levels, as well as stimulate osteoblast proliferation and osteoclast apoptosis [123]. *E. longifolia* has been shown recently to protect against bone calcium loss in orchidectomised rats, the model for androgen-deficient osteoporosis. Supplementation with it extract elevated the testosterone levels, reduced the bone resorption marker and upregulated OPG gene expression of the orchidectomised rats. These actions may be responsible for the protective effects of *E. longifolia* extract against bone resorption due to androgen deficiency [182]. Further studies on the regulation of OPG production by *E. longifolia* may provide insight into this novel mechanism. *E. longifolia* exerts proandrogenic effects that enhance the testosterone level, as well as stimulate osteoblast proliferation and osteoclast apoptosis. This will maintain bone remodelling activity and reduce bone loss. Phytochemical components of *E. longifolia* may also prevent osteoporosis via its antioxidative property. Hence, *E. longifolia* has the potential as a complementary treatment for male osteoporosis [123].

### 5.9. Miscellaneous Effects

#### 5.9.1. Hormonal Effects

A standardized extract of *E. longifolia* Jack containing a high concentration of quassinoids (20% eurycomanone and 4% 13α,21-dihydroeurycomanone) had antiestrogenic effects against 17α-ethynylestradiol (EE)-induced uterotrophy of immature rats [86]. Another study showed that the *E. longifolia* plant extract normalized irregular estrous cycles and reduced the follicular morphological damage caused by chronic testosterone administration in the female rats. The reversal effect derived from the anti-estrogenic properties of the plant quassinoids. Further work is required to identify the exact mechanism behind the ameliorative effects of *E. longifolia* [183].

#### 5.9.2. Ergogenic Effects

The ergogenic effects of *E. longifolia* were discussed in a review [184]. The authors reviewed its medicinal properties and studies investigating physiological responses and endurance exercise performance. Increased testosterone, as shown in animal models [115], has been suggested in anecdotal reports as being responsible for *E.* *longifolia*-induced increases in muscle mass and strength in humans. According to secondary sources, *E. longifolia* enhances testosterone production by the Leydig cells and frees bound testosterone for use by muscles [185].

#### 5.9.3. Insecticidal Effects

*E. longifolia*-containing smoke from mosquito coils resulted in increased knock-down activities of mosquitos, but not increased mortality [186]. One study showed that *E. longifolia* exhibits the highest anti-protozoal activity at 1.0 mg/mL. The ethyl acetate fraction exhibited a slightly higher percentage of anti-protozoal activity and demonstrated the highest anti-protozoal activity against *Blastocystis* sp. isolates and showed a sizeable reduction in the cell count in comparison to the allopathic drugs [187].

#### 5.9.4. Muscular Effects

In animal research, *E. longifolia* extracts increased weight of the levator ani muscle (involved in tail wagging) in castrated animals, but not testosterone-treated animals and uncastrated animals [188].

#### 5.9.5. Antiulcer Effect

A bioassay study of *Pasak bumi (E. longifolia)* led to the isolation of four quassinoids, pasakbumin-A, -B, -C, and -D. Both pasakbumin-A (eurycomanone) and pasakbumin-B exhibited potent antiulcer activity [50]. In one other study, Qodriyah *et al.*, investigation showed that *E. longifolia* in Radix is as effective as ranitidine in the treatment of ethanol-induced gastric lesions in rats [189].

#### 5.9.6. Anti-Rheumatism Effect

Studies showed that decoction, and an alcoholic extract of *E. longifolia* roots are used to treat rheumatism [45,190].

## 6. Pharmacokinetics

### 6.1. Absorption

The bioavailability of the constituent eurycomanone was investigated in animal research [65]. Following intravenous injection, eurycomanone was detected in the plasma, declining to zero within 8 h. Following oral administration, C_max_ and T_max_ values were 0.33 ± 0.03 mcg/mL and 4.40 ± 0.98 h, respectively. The plasma concentration was lower following oral administration *vs.* intravenous administration, even at a much higher oral dose (five times the dose). The authors concluded that eurycomanone is poorly bioavailable orally (10.5%).

In animal research, less than 1% of the constituent 9-methoxycanthin-6-one was found to be absorbed orally [100].

Following oral administration of a standardized extract (Fr 2) of *E. longifolia*, 13 α(21)-epoxy-eurycomanone had a higher C_max_ than eurycomanone (1.61 ± 0.41 mcg/mL *vs.* 0.53 ± 0.10 mcg/mL) [62,86,191]. The absolute bioavailability was also higher due to increased membrane permeability (higher log Kow value of −0.43 *vs.* −1.46 at pH 1). Following oral administration of a standardized extract (Fr 2) of *E. longifolia*, eurycomanol and 13α,21-dihydroeurycomanone were not detected in plasma [62].

### 6.2. Distribution

In animal research, the volume of distribution (*V_d_*) of eurycomanone was relatively high (0.68 ± 0.30 L/kg), suggesting that it is well distributed in the extravascular fluids [65].

### 6.3. Excretion

Following intravenous injection, the mean elimination rate constant (*k_e_*) and clearance (*CL*) for eurycomanone were 0.88 ± 0.19 h^−1^ and 0.39 ± 0.08 L/h/kg, respectively [65].

### 6.4 CYP Inhibition

*In vitro* evaluation of the modulatory effects of eurycomanone, an active constituent of *E. longifolia* on cytochrome P450 (CYP) isoforms CYP1A2, CYP2A6, CYP2C8, CYP2C9, CYP2C19, CYP2E1 and CYP3A4 were conducted by Pan *et al.* They indicated that eurycomanone did not potently inhibit any of the investigated CYP isoforms, with IC50 values greater than 250 μg/mL, hence appears to be little likelihood of drug-herb interaction via CYP inhibition [192].

Recent, CYP inhibition study of *E.* *longifolia* by Han *et al.*, showed that *E. logifolia* has a weak, concentration-dependent inhibitory effect on CYP1A2, CYP2A6, and CYP2C19 isozymes, showing IC_50_ values of 324.9, 797.1, and 562.9 μg/mL, respectively. It needs careful attention in taking *E.* *longifolia* extracts products with conventional drugs [193].

### 6.5 Half-Life

Following intravenous administration of a standardized extract (Fr. 2) of *E. longifolia*, 13α(21)-epoxyeurycomanone had a longer biological half-life than eurycomanone (0.75 ± 0.25 h *vs.* 0.35 ± 0.04 h), due to a lower elimination rate constant [62]. Conversely, another study reported the biological half-life (t_1/2_) of eurycomanone to be 1.00 ± 0.26 h [65].

## 7. Evidence-Based Toxicology

### 7.1. Safety and Toxicity

Although *E. longifolia* has been used in traditional medicine for generations in Malaysia, it was only in the late 1990s that researchers started to pay more attention to its safe dosage and toxicity profile. Safety studies carried out thus far showed that Tongkat Ali (*E. longifolia*) concentrations used therapeutically (2.5 µg·mL^−1^) appear not to have any detrimental effects on human spermatozoa *in vitro* [194]. However, at concentrations higher than 100 µg·mL^−1^, cytotoxic effects might occur [36,160] supporting *in vivo* data by Tambi and Kadir, that the extract is not toxic [9]. In animal studies, no negative effect on the offspring could be found, either in terms of malformations or of any effect on body weight or the number of the offspring [124]; yet an acute toxicity study done by Satayavivad *et al.* has found that the oral Lethal Dose 50 (LD_50_) of the alcoholic extract of *E. longifolia* in mice is between 1500–2000 mg/kg, while the oral LD_50_ of the aqueous extract form is more than 3000 mg/kg [194]. These authors further showed that dosages of 200 mg·kg^−1^ body weight of the ethanolic extract and 300 mg·kg^−1^ of the aqueous extract daily were not toxic. Only at dosages above 1200 mg·kg^−1^ body weight, were significant hepatotoxic effects shown in the rat [195]. The acute toxicity studies in mice found that the n-butanol fraction of *E. longifolia* was the most toxic, mainly due to eurycomanone [191].

It simply means that as the composition of ethanolic, n-butanolic- and aqueous-based fractions of *E. longifolia* differs, therefore, LD_50_ as well daily effective doses are also varied among fractions. The water-based fraction of *E. longifolia* is considered the safest among others, as its LD_50_ value is comparatively high (>3000 mg/kg) than other fractions, so this needs attention when using different fractions of *E. longifolia* and proper reference of the corresponding range of LD_50_.

Choudhary *et al.*, investigated the acute, subacute and subchronic toxicity of the standardized aqueous *E. longifolia* extract (Physta^®^) in a rat model. Male and female Wistar rats were treated for 90 days with *E. longifolia* concentrations from 250 mg·kg^−1^ body weight to 2000 mg·kg^−1^ body weight. Results clearly show no significant changes in blood chemistry and haematological parameters. There were also no histopathological changes and even in acute toxicity tests, no changes in mortality or in the behaviour of the animals was seen [196].

With reference to the prostate, the Endocrine Society recommends that prostate cancer (PCa) has to be regarded as a contraindication for any testosterone treatment [197]. Considering that *E. longifolia* extract increases the serum testosterone concentrations, there might be a potential risk from its treatment in elderly men, which might cause prostatic problems. On other hands, a randomized double-blind, placebo-controlled clinical trial by Ismail *et al.*, revealed no difference between the placebo and the verum group for serum prostate-specific antigen (PSA) levels [114]. Li *et al.* showed that neither mutagenicity nor clastogenicity was noted, and the acute oral LD_50_ was more than 6 g/kg b.w for *E. longifolia* extract. After 4-week subacute and 13-week subchronic exposure paradigms (0, 0.6, 1.2, and 2 g/kg b.w. per day), adverse effects attributable to test compound was not observed with respect to body weight, hematology, serum biochemistry, urinalysis, macropathology, or histopathology. However, the treatment significantly reduced prothrombin time, partial thromboplastin time, blood urea nitrogen, creatinine, aspartate aminotransferase, creatine phosphate kinase, lactate dehydrogenase, and cholesterol levels, especially in males (*p* < 0.05). Calculated acceptable daily intake (ADI) for *E. longifolia* extract, was up to 1.2 g/adult/day. The investigated intension of *E. longifolia* extract intake by Li *et al.* was to calculate its safety profile in health supplements. This information is useful for product development and safety management [198].

From Hamoud and Qamar’s findings, it is strongly suggested that *E. longifolia* Jack has no evidence of side effects or any deleterious effect on the pancreatic tissues when used orally in small quantities for more than a month. Regular *E. longifolia* use at low doses does not appear to cause any toxic effect on the pancreas and could be considered a safe herbal supplement as far as the safety of the pancreas in human beings is concerned [199].

No toxic symptoms were observed in TAF273-treated pregnant female rats, and their pregnancies were normal with no fetus abnormalities. After administration of a 100 mg/kg daily dose of TAF273, which is almost >10-fold lower than the LD_50_ value, no adverse effect was observed in reproductive toxicity and teratology studies in rats. The authors concluded that any human dose derived from converting the rat doses of 100 mg/kg/day or below, may be considered safe for further clinical studies [200].

Chen *et al.*, investigated the effects of standardized water extract of *E. longifolia* the Physta^®^ at dose of 400 mg/day for 6 weeks, showed no significant changes in both the liver and renal functions tests, so supplementation of *E. longifolia* at this dosage and duration was non-toxic to the liver and renal functions [117].

The Food and Drug Administration has suggested that the extrapolation of animal doses to human doses is correctly performed only through normalization to BSA, which often is represented in mg/m^2^. The human dose equivalent can be more appropriately calculated by using the formula as HED (mg/kg) = Animal dose (mg/kg) [Animal *Km*/Human *Km*] [201].

*E. longifolia* is considered safe as long as it is not taken in a high dose. Based on the results of previous toxicity studies, *E. longifolia* is normally recommended to be administered to men at the dose of 200–400 mg daily and should be used with caution, especially in the elderly. Currently, *E. longifolia* is commercially sold worldwide following this established dosage in the form of tablets for easier daily consumption [195].

### 7.2. Precautions/Contraindications

Based on studies in animals suggesting that *E. longifolia* reduced blood glucose in hyperglycemic animals [168] and unpublished studies in humans suggesting the possibility for increased blood glucose, it should be used cautiously in patients using hypoglycemic agents. Also use with in individuals using propranolol, as in healthy males, a water-based extract of *E. longifolia* decreased the bioavailability of propranolol [202].

It should be used cautiously in people with weakened immune systems, as some evidence suggests that it may further weaken immune function, according to secondary sources [185]. Use is to be avoided in individuals with diseases like breast cancer, prostate cancer, heart disease, kidney disease, liver disease, or sleep apnea, according to secondary sources [185,203].

Use in patients with known allergy or hypersensitivity to *E. longifolia*, its constituents, or hypersensitivity to other members of the Simaroubaceae family is also to be avoided [185,203]. Use during pregnancy and lactation and in children is not suggested due to a lack of sufficient data [185]. Information on *E. longifolia’s* effects on lactation is lacking in the National Institute of Health’s Lactation and Toxicology Database (LactMed) [203].

One *in vivo* study indicates that in animals, no negative effect on the offspring could be found, neither in terms of malformations nor of any effect on body weight or the number of the offspring [195]. Low *et al.*, investigated reproductive toxicity, up-&-down acute toxicity, and two generations of fetus teratology in orally TAF273 (quassinoid-rich *E.* *longifolia* extract)-treated rats. The results showed that the lethal dose (LD_50_) of TAF273 for male and female rats was >2000 and 1293 mg/kg, respectively. Fertility index and litter size of the treated rats were significantly increased, compared to non-treated rats [200].

## 8. Conclusions

Novel molecular diversity (abbreviate as NMD) poses a formidable challenge for a rational drug design. Bioassay-guided fractionation of natural products is one high-throughput screening (HTS) approach to identify potent bioactive molecules. Today natural products continue to play a major role as active substances and model molecules for the discovery and validation of drug targets. Herbal medicines have been used for thousands of years in almost all developing countries and recently, the World Health Organization estimated that 80% of people worldwide rely on herbal medicines for some part of their primary health care. A multidisciplinary approach in new drug discovery, mostly involving the generation of truly novel molecular diversity from natural herbal sources, combined with combinatorial synthetic methodologies, provides the best solution to increase the novelty and productivity in novel drug discovery and further development. Screening for new drugs in plant sources implies the screening of extracts for the presence of novel compounds as well as investigation of their biological activities.

Whereas over 100,000 secondary metabolites are already known, only a small percentage of all species have been studied for the presence of secondary metabolites. It is currently estimated that approximately 420,000 plant species exist in Nature [204], and less than 5% of known plants have been screened for one or more biological activities [205].

The advances in the phytochemical analysis, especially the impact of high-performance liquid chromatography (HPLC)-coupled spectroscopy on natural product research, have been tremendous in the rapid characterization of natural product extracts. The concerted use of photodiode-array UV-Vis absorbance detection (DAD), MS and even NMR spectroscopy, LC-DAD, -MS and -NMR has opened entirely new possibilities to characterize the profiles of the metabolites in the biological extracts [206]. MS-guided isolation has taken great progress in drug discovery. Rapid processes are required for post-HTS “hit” characterization, at which point milligram or more quantities of the compound of interest must typically be isolated for further biological evaluation, as well as complete structure elucidation that illustrates the complementary nature of NMR and MS data for phytochemical analysis. Several *in vitro* tests that illuminate the property of interest are available for screening plants and their constituents in order to find the most effective materials and components for further investigations [207].

*E. longifolia* Jack is reported to be rich in various classes of bioactive compounds such as quassinoids, canthin-6-one alkaloids, β-carboline alkaloids, triterpene tirucallane type, squalene derivatives and biphenyl neolignans, eurycolactone, laurycolactone, and eurycomalactone, and bioactive steroids. LC-MS is also recognized as a powerful tool for identification and quantification of various major and minor constituents from *E. longifolia*, which is used as a folk medicine for sexual dysfunction, aging, malaria, cancer, diabetes, anxiety, aches, constipation, exercise recovery, fever, increased energy, increased strength, leukemia, osteoporosis, stress, syphilis and glandular swelling; it is also used as an aphrodisiac, antibacterial, appetite stimulant and health supplement.

It is suggested that the integration of natural chemistry, medicinal chemistry, biology, pharmacology, toxicology and other associated disciplines could be the most promising way to discovering drugs and to ensure a greater chance of advancing natural products and natural-based products into therapeutically useful drugs.

*E.* *longifolia* is one of the most useful and safe traditional herbal medicines. Based on established literature on the health benefits of *E.* *longifolia*, it is important to focus more attention on its more active constituents and these constituents’ identification, determination, further development and most importantly, standardization. Besides the available data, more evidence regarding its therapeutic efficacy and safety is required, to establish proper clinical recommendations for *E. longifolia*’s safe use. By doing so, it is not hard to imagine that not far into the future *E.* *longifolia* will be considered a rich source for new drug candidates. It is also very important to conserve this valuable medicinal plant for the health benefit of future generations.

## Figures and Tables

**Table 1:** Major isolated chemical constituents with metabolites from *Eurycoma longifolia* Jack and their pharmacological effects.

Chemical Compounds Isolated	Plant Parts	Pharmacological Effects	References (Isolation & Pharmacological Effects)
Eurycomanone (C_20_) 13α,21-Dihydroeurycomanone 13α(21)-Epoxyeurycomanone 13β-Methyl,21-dihydroeurycomanone 12-Acetyl-13,21-dihydoeurycomanone 15-Acetyl-13α(21)-epoxyeurycomanone 12,15-Diacetyl-13α(21)-epoxyeurycomanone 1β,12α,15β-Triacetyleurycomanone	Roots	Increased testosterone production Improved spermatogenesis Expression Suppression of lung cancer cell tumor markers, prohibitin, annexin 1 and endoplasmic reticulum protein 28 Cytotoxicity against human lung cancer (A-549), and human breast cancer (MCF-7) cell lines Antimalarial against *P. falciparum* NF-κB inhibitor Anti-estrogenic activity	[36,39,45,51,58,59,60,61,62,63,64,65,66,67,68]
Eurycomanol (C_20_) Eurycomanol-2-*O*-β-d-glucoside 13β,18-Dihydroeurycomanol 13β,21-Dihydroxyeurycomanol	Roots	Antimalarial against *P. falciparum*	[36,39,48,52,58,59,60,64,66,67]
5α,14β,15β-Trihydroxyklaineanone 11-Dehydroklaineanone 12-epi-11-Dehydroklaineanone 14,15β-Dihydroxyklaineanone 15β-Hydroxyklaineanone 15β-Acetyl-14-hydroxyklaineanone	Leaves, Roots	Cytotoxicity against human lung cancer (A-549), and human breast cancer (MCF- 7) cell lines NF-κB inhibitor	[35,36,45,48,51,58,69]
Laurycolactones A and B (C_18_)	Roots	Cytotoxicity against human HT1080	[42,69]
Eurycomalactone (C_19_) 6α-Hydroxyeurycomalactone 7α-Hydroxyeurycomalactone 5,6-Dehydroeurycomalactone Eurycomadilactone (C_20_) 5-iso-Eurycomadilactone 13-*epi*- Eurycomadilactone	Roots	Cytotoxicity against human lung cancer (A-549), breast cancer (MCF- 7) and gastric cancer (MGC-803) cell lines Cytotoxicity against human HT1080 cells Antimalarial against *P. falciparum*	[36,45,49,51,58,59,61,69,70]
Eurycomalides A and B (C_19_) Eurycomalide C Eurycomalide D Eurycomalide E	Roots	Cytotoxicity against human lung cancer (A-549), and human breast cancer (MCF-7) cell lines NF-κB inhibitor	[36,42,45]
Eurycomaoside	Roots	ENR	[71]
Longilactone (C_19_) 6-Dehydroxylongilactone 11-Dehydroklaineanone	Leaves, Roots	Cytotoxicity against human HT1080 Cytotoxicity against human lung cancer (A-549), and human breast cancer (MCF-7) cell lines Compounds possess anti-tumor promoting, antischistosomal and plasmodicidal activities NF-κB inhibitor	[36,42,45,58,69,72,73]
Eurycolactone A(C_20_) Eurycolactone B(C_18_) Eurycolactone D (C_18_) Eurycolactones E, F (C_19_)	Roots	Cytotoxicity against human HT1080 NF-κB inhibitor	[42,44,45,51,74]
Eurylactones A and B (C_18_) Eurylactones E, F and G (C_19_)		ENR	[51,69,75]
Canthin-6-one alkaloids 9-Methoxycanthin-6-one 9-Hydroxycanthin-6-one 9-Methoxycanthin-6-one*-N-*oxide 9-Hydroxycanthin-6-one-*N-*oxide 1-Hydroxy-9-methoxycanthin-6-one 5-Hydroxymethyl-9-methoxycanthin-6- 10-Hydroxycanthin-6-one 10-Hydroxy-9-methoxycanthin-6-one 10-Hydroxy-11-methoxycanthin-6-one 11-Hydroxy-10-methoxycanthin-6-one 4,9-Dimethoxycanthin-6-one 5,9-Dimethoxycanthin-6-one 9,10-Dimethoxycanthin-6-one 9-Methoxy-3-methylcanthin-5,6-dione	Plant (bark, Stem and Roots)	Oxidative burst inhibitory, and cytotoxic activity Cytotoxicity against human lung cancer (A-549), and human breast cancer (MCF-7) cell lines Antimalarial against *P. falciparum* Anti-ulcer activity NF-κB inhibitor Active cytotoxicity against human cancer cell types (breast, colon, fibrosarcoma, lung, melanoma, KB) and murine lymphocytic leukemia (P-388)	[36,45,62,69,76,77,78,79,80,81]
β-Carboline alkaloids 7-Hydroxy-β-carboline-1-propionic acid 1-Methoxymethyl-β-carboline *n* -pPentyl β-carboline-1-propionate β-Cararboline-1-propionic acid β-7-Methoxycarboline-1-propionic acid	Roots	Antimalarial against *P. falciparum* Anti-inflammatory effect via NF-κB inhibition	[56,61,76,82]
Biphenyl neolignans 2-Hydroxy-3,2-dimethoxy-4-(2,3-epoxy-1-hydroxypropyl)-5-(3-hydroxy-1-propenyl)-biphenyl 2-Hydroxy-3,2,6-trimethoxy-4-(2,3-epoxy-1-hydroxypropyl)-5-(3-hydroxy-1-propenyl)-biphenyl	Stem	ENR	[47,55]
Squalene-type triterpenes Eurylene 14-Deacetyleurylene Longilene peroxide Teurilene	Stem	Cytotoxicity Cytotoxic activity against KB cells	[54,83,84]
Phytosterols (Campesterol, stigmasterol, sitosterol) Saponins	Plant	ENR	[85]
Pasakbumin-A, -B, -C, -D (C_20_)	Roots	Anti-ulcer Cytotoxicity against human lung cancer (A-549) and human breast cancer (MCF-7) cell lines	[36,50]
Tirucallane-type triterpenes (Niloticin, dihydroniloticin, piscidinol A, bourjotinolone A, 3-episapelin A, melianone, and hispidone)	Stem	Anti-cancer activity against ovarian leukemia and renal cell lines	[69]
Tirucallane-type triterpenoid 23,24,25-Trihydroxytirucall-7-en-3,6-dione	Stem	ENR	[77]
Oxasqualenoid	Stem	ENR	[77]
Anthraquinones and anthraquinone glucosides	Roots	ENR	[78]
Glycoprotein	Plant	ENR	[86]
In cell suspension cultures, two canthin-6-one alkaloids 9-Hydroxycanthin-6-one 9-Methoxycanthin-6-one	Plant	Antimalarial against *P. falciparum*	[76,87,88,89]
Predominant amino acids Alanine, proline, arginine, and serine	Plant (Roots)	ENR	[90]
A 4.3kDa bioactive peptide	Roots	ENR	[91]
Starch (about 39%)	Roots	ENR	[92]

Note: ENR = Evidence Not Reported (much of the available evidence about the pharmacological effects of *Eurycoma Longifolia*, is related to its extracts (mixtures), so these effects cannot be correlated with specific chemical constituents or groups).

## References

[1] Bodeker G., Ong C.K. (2005). WHO Global Atlas of Traditional, Complementary and Alternative Medicine.

[2] WHO (2002). Traditional Medicine Strategy 2002–2005.

[3] Lancet J. (2003). Herbal remedies and the bias against Ayurveda. Curr. Sci..

[4] Duraz A.Y., Khan S.A. (2011). Knowledge, attitudes and awareness of community pharmacists towards the use of herbal medicines in muscat region. Oman Med. J..

[5] Patwardhan B., Vaidya A.D., Chorghade M. (2004). Ayurveda and natural products drug discovery. Curr. Sci. Bangalore.

[6] Fabricant D.S., Farnsworth N.R. (2001). The value of plants used in traditional medicine for drug discovery. Environ. Health Perspect..

[7] Bhat R., Karim A. (2010). Tongkat Ali (*Eurycoma longifolia* Jack): A review on its ethnobotany and pharmacological importance. Fitoterapia.

[8] AbdRahman K., Niiyama K., Azizi R., Appanah S., Iida S. (2002). Species assembly and site preference of tree species in a primary seraya-ridge forest of Peninsular Malaysia. J. Trop. For. Sci..

[9] Sulaiman B., Jaafar A., Mansor M. (1990). Some medicinal plants from Sungai Kinchin, Pahang, Malaysia. Malay. Nat. J..

[10] Chua G., Koh B., Lau S., Lee S., Mathias M., Turner I., Yong J. (1995). The nutrient status of the plateau heath forest on Gunung Keriong, Pahang, Peninsular Malaysia. J. Trop. For. Sci..

[11] Chua L., Kamarudin S., Markandan M., Hamidah M. (2005). A preliminary checklist of vascular plants from the Machinchang Range, Pulau Langkawi, Peninsular Malaysia. Malay. Nat. J..

[12] Ang H., Ikeda S., Gan E. (2001). Evaluation of the potency activity of aphrodisiac in *Eurycoma longifolia* Jack. Phytother. Res..

[13] Kulip J. (2009). Medicinal plants of Sabah, Malaysia: Potential for agroforestry. JIRCAS Work. Rep..

[14] Adenan M.I. (1999). Opportunities on the planting of medicinal and herbal plants in Malaysia. Planter.

[15] Mohidin A., Tajudin M.H., YuShyun C., Mohtar M., Subramaniam V., Yunos N. (2002). Sustainable production of medicinal plants through cultivation: The golden hope experience, towards modernisation of research and technology in herbal industries. Proceedings of the Seminar on Medicinal and Aromatic Plants.

[16] Group H.M.R. (2002). Compendium of Medicinal Plants used in Malaysia. Kuala Lumpur Instit. Med. Res. Malays..

[17] Tambi M., Kadir A. (2006). *Eurycoma Longifolia* jack: A potent adaptogen in the form of water-soluble extract with the effect of maintaining men’s health. Asian J. Androl..

[18] Keng H. (1978). Orders and Families of Malayan Seed Plants.

[19] Keng H., Keng R.S.L. (1990). The Concise Flora of Singapore: GYMNOSPERMS and Dicotyledons.

[20] Goh S.H., Chuah C., Mok J., Soepadmo E. (1995). Malaysian Medicinal Plants for the Treatment of Cardiovascular Diseases.

[21] Osman A., Jordan B., Lessard P.A., Muhammad N., Haron M.R., Riffin N.M., Sinskey A.J., Rha C., Housman D.E. (2003). Genetic diversity of Eurycoma longifolia inferred from single nucleotide polymorphisms. Plant Physiol..

[22] Tnah L.H., Lee C.T., Lee S.L., Ng K.K.S., Ng C.H., San Hwang S. (2011). Microsatellite markers of an important medicinal plant, *Eurycoma longifolia* (Simaroubaceae), for DNA profiling. Am. J. Bot..

[23] Razi A.R.M., Abdul-Aziz A., Alwee S.S.B.S., Aziz R. (2013). Relationships between Malaysians Cultivars of Tongkat Ali (*Eurycoma Longifolia* Jack) Obtained through RAPD Analysis. Int. J. Biotechnol. Well. Ind..

[24] Aziz S., Akeng G., Kandasamy K. (2000). Induction of somatic embryos from cotyledonary tissue of Tongkat Ali (*Eurycoma longifolia*). J. Trop. Med Plants.

[25] Danial M., Keng C.L., Alwee S.S.R.S., Subramaniam S. (2005). Seed histology of recalcitrant *Eurycoma longifolia* plants during germination and its beneficial attribute for hairy roots production. J. Med. Plants Res..

[26] Hasnida H., Aziah M., Salbiah M., Fadhilah Z., Haliza I., Mohamed A.H., Parlan I.H., Ibrahim S., Safiah Yusmah M., Muhammed Azmi M. (2001). Multiplication of Shoots from in Vitro Germinated Seedlings of *Eurycoma longifolia* and *Aquilaria malaccensis*, tropical forestry research in the new millennium: Meeting demands and challenges. Proceedings of the International Conference on Forestry and Forest Products Research (CFFPR 2001).

[27] Hussein S., Ibrahim R., Kiong A.L.P. (2006). Adventitious shoots regeneration from root and stem explants of *Eurycoma longifolia* Jack-an important tropical medicinal plants. Int. J. Agric. Res..

[28] Hussein S., Ibrahim R., Kiong A.L.P., Daud S.K. (2005). Micropropagation of *Eurycoma longifolia* Jack via formation of somatic embryogenesis. Asian J. Plant Sci..

[29] Mahmood M., Normi R., Subramaniam S. (2010). Optimization of Suitable Auxin Application in a Recalcitrant Woody Forest Plant of *Eurycoma Longifolia* (Tongkat Ali) for Callus Inducation. Afr. J. Biotechnol..

[30] Siregar L., Keng C. (2002). *In vitro* shoot organogenesis of *Eurycoma longifolia*. Planter.

[31] Sobri H., Marziah M., Azizol A., YuShyun C., Mohtar M., Subramaniam V., Yunos N. (2002). Tissue Culture of Tongkat Ali (*Eurycoma longifolia*) for Mass Production, towards modernisation of research and technology in herbal industries. Proceedings of the Seminar on Medicinal and Aromatic Plants.

[32] Ling A.P.K., Phua G.A.T., Tee C.S., Hussein S. (2010). Optimization of protoplast isolation protocols from callus of *Eurycoma longifolia*. J. Med. Plants Res..

[33] Lulu T., Park S.Y., Ibrahim R., Paek K.Y. (2015). Production of biomass and bioactive compounds from adventitious roots by optimization of culturing conditions of *Eurycoma longifolia* in balloon-type bubble bioreactor system. J. Biosci. Bioeng..

[34] Jamal J.A. (2006). Malay traditional medicine. Tech. Monit..

[35] Jiwajinda S., Santisopasri V., Murakami A., Hirai N., Ohigashi H. (2001). Quassinoids from *Eurycoma longifolia* as plant growth inhibitors. Phytochemistry.

[36] Kuo P.C., Damu A.G., Lee K.H., Wu T.S. (2004). Cytotoxic and antimalarial constituents from the roots of Eurycoma longifolia. Biorg. Med. Chem..

[37] Hussein S., Ibrahim R., LingPick K. (2007). A summary of reported chemical constituents and medicinal uses of Eurycoma longifolia. J. Trop. Med. Plants.

[38] Chan K., Lee S., Sam T., Han B. (1989). A quassinoid glycoside from the roots of *Eurycoma longifolia*. Phytochemistry.

[39] Darise M., Kohda H., Mizutani K., Tanaka O. (1982). Eurycomanone and eurycomanol, quassinoids from the roots of *Eurycoma longifolia*. Phytochemistry.

[40] Fiaschetti G., Grotzer M., Shalaby T., Castelletti D., Arcaro A. (2010). Quassinoids: From traditional drugs to new cancer therapeutics. Curr. Med. Chem..

[41] Grieco P.A., Morre D.M. (1998). Mode of action of the anticancer quassinoids—Inhibition of the plasma membrane NADH oxidase. Life Sci..

[42] Miyake K., Tezuka Y., Awale S., Li F., Kadota S. (2009). Quassinoids from *Eurycoma longifolia*. J. Nat. Prod..

[43] Mahfudh N., Pihie A.H.L. (2008). Eurycomanone induces apoptosis through the up-regulation of p53 in human cervical carcinoma cells. J. Cancer Mol..

[44] Ang H.H., Hitotsuyanagi Y., Takeya K. (2000). Eurycolactones A–C, novel quassinoids from *Eurycoma longifolia*. Tetrahedron Lett..

[45] Tran T.V.A., Malainer C., Schwaiger S., Atanasov A.G., Heiss E.H., Dirsch V.M., Stuppner H. (2014). NF-κB Inhibitors from *Eurycoma longifolia*. J. Nat. Prod..

[46] Athimulam A., Kumaresan S., Foo D.C.Y., Sarmidi M.R., Aziz R. (2006). Modelling and Optimization of *Eurycoma longifolia* Water Extract Production. Food Bioprod. Process..

[47] Chua L.S., Amin N.A.M., Neo J.C.H., Lee T.H., Lee C.T., Sarmidi M.R., Aziz R.A. (2011). LC-MS/MS-based metabolites of *Eurycoma longifolia* (Tongkat Ali) in Malaysia (Perak and Pahang). J. Chromatogr. B.

[48] Chan K., Lee S., Sam T., Tan S., Noguchi H., Sankawa U. (1991). 13β,18-dihydroeurycomanol, a quassinoid from *Eurycoma longifolia*. Phytochemistry.

[49] Chan K., Iitaka Y., Noguchi H., Sugiyama H., Saito I., Sankawa U. (1992). 6α-Hydroxyeurycomalactone, a quassinoid from *Eurycoma longifolia*. Phytochemistry.

[50] Tada H., Yasuda F., Otani K., Doteuchi M., Ishihara Y., Shiro M. (1991). New antiulcer quassinoids from *Eurycoma longifolia*. Eur. J. Med. Chem..

[51] Park S., Nhiem N.X., Van Kiem P., Van Minh C., Tai B.H., Kim N., Yoo H.H., Song J.H., Ko H.J., Kim S.H. (2014). Five new quassinoids and cytotoxic constituents from the roots of *Eurycoma longifolia*. Bioorg. Med. Chem. Lett..

[52] Meng D., Li X., Han L., Zhang L., An W., Li X. (2014). Four new quassinoids from the roots of *Eurycoma longifolia* Jack. Fitoterapia.

[53] Itokawa H., Kishi E., Morita H., Takeya K., Iitaka Y. (1991). Eurylene, a new squalene-type triterpene from *Eurycoma longifolia*. Tetrahedron Lett..

[54] Morita H., Kishi E., Takeya K., Itokawa H., Iitaka Y. (1993). Squalene derivatives from *Eurycoma longifolia*. Phytochemistry.

[55] Morita H., Kishi E., Takeya K., Itokawa H. (1992). Biphenylneolignans from wood of *Eurycoma longifolia*. Phytochemistry.

[56] Mitsunaga K., Koike K., Tanaka T., Ohkawa Y., Kobayashi Y., Sawaguchi T., Ohmoto T. (1994). Canthin-6-one alkaloids from *Eurycoma longifolia*. Phytochemistry.

[57] Choo C.Y., Chan K.L. (2002). High performance liquid chromatography analysis of canthinone alkaloids from *Eurycoma longifolia*. Planta Med..

[58] Chan K.L., Choo C.Y., Morita H., Itokawa H. (1998). High performance liquid chromatography in phytochemical analysis of *Eurycoma longifolia*. Planta Med..

[59] Udani J.K., George A.A., Musthapa M., Pakdaman M.N., Abas A. (2014). Effects of a proprietary freeze-dried water extract of *Eurycoma longifolia* (Physta) and Polygonum minus on sexual performance and well-being in men: A randomized, double-blind, placebo-controlled study. Evid. Based Complement. Altern. Med..

[60] Chan K.L., O'Neill M.J., Phillipson J.D., Warhurst D.C. (1986). Plants as Sources of Antimalarial Drugs. Part 31 *Eurycoma longifolia*. Planta Med..

[61] Kardono L.B., Angerhofer C.K., Tsauri S., Padmawinata K., Pezzuto J.M., Kinghorn A.D. (1991). Cytotoxic and antimalarial constituents of the roots of *Eurycoma longifolia*. J. Nat. Prod..

[62] Low B.S., Teh C.H., Yuen K.H., Chan K.L. (2011). Physico-chemical effects of the major quassinoids in a standardized *Eurycoma longifolia* extract (Fr 2) on the bioavailability and pharmacokinetic properties, and their implications for oral antimalarial activity. Nat. Prod. Commun..

[63] Wernsdorfer W.H., Ismail S., Chan K.L., Congpuong K., Wernsdorfer G. (2009). Activity of *Eurycoma longifolia* root extract against Plasmodium falciparum *in vitro*. Wien. Klinische Wochenschr..

[64] Ang H.H., Chan K.L., Mak J.W. (1995). Effect of 7-day daily replacement of culture medium containing *Eurycoma longifolia* Jack constituents on the Malaysian Plasmodium falciparum isolates. J. Ethnopharmacol..

[65] Low B.S., Ng B.H., Choy W.P., Yuen K.H., Chan K.L. (2005). Bioavailability and pharmacokinetic studies of eurycomanone from *Eurycoma longifolia*. Planta Med..

[66] Ang H.H., Chan K.L., Mak J.W. (1995). *In vitro* antimalarial activity of quassinoids from *Eurycoma longifolia* against Malaysian chloroquine-resistant Plasmodium falciparum isolates. Planta Med. J. Med. Plant Res..

[67] Darise M., Kohda H., Mizutani K., Tanaka O. (1983). Revision of configuration of the 12-hydroxyl group of eurycomanone and eurycomanol, quassinoids from *Eurycoma longifolia*. Phytochemistry.

[68] Wong P.F., Cheong W.F., Shu M.H., Teh C.H., Chan K.L., AbuBakar S. (2012). Eurycomanone suppresses expression of lung cancer cell tumor markers, prohibitin, annexin 1 and endoplasmic reticulum protein 28. Phytomedicine.

[69] Itokawa H., Qin X.-R., Morita H., Takeya K. (1993). C18 and C19 quassinoids from *Eurycoma longifolia*. J. Nat. Prod..

[70] Ang H., Lee K. (2002). Effect of *Eurycoma longifolia* Jack on orientation activities in middle-aged male rats. Fundam. Clin. Pharmacol..

[71] Bedir E., Abou-Gazar H., Ngwendson J.N., Khan I.A. (2003). Eurycomaoside: A new quassinoid-type glycoside from the roots of *Eurycoma longifolia*. Chem. Pharm. Bull..

[72] Morita H., Kishi E., Takeya K., Itokawa H., Iitaka Y. (1993). Highly oxygenated quassinoids from *Eurycoma longifolia*. Phytochemistry.

[73] Jiwajinda S., Santisopasri V., Murakami A., Kawanaka M., Kawanaka H., Gasquet M., Eilas R., Balansard G., Ohigashi H. (2002). *In vitro* anti-tumor promoting and anti-parasitic activities of the quassinoids from *Eurycoma longifolia*, a medicinal plant in Southeast Asia. J. Ethnopharmacol..

[74] Ang H.H., Hitotsuyanagi Y., Fukaya H., Takeya K. (2002). Quassinoids from *Eurycoma longifolia*. Phytochemistry.

[75] Itokawa H., Kishi E., Morita H., Takeya K. (1992). Cytotoxic quassinoids and tirucallane-type triterpenes from the woods of *Eurycoma longifolia*. Chem. Pharm. Bull..

[76] Kuo P.C., Shi L.S., Damu A.G., Su C.R., Huang C.H., Ke C.H., Wu J.B., Lin A.J., Bastow K.F., Lee K.H. (2003). Cytotoxic and antimalarial β-carboline alkaloids from the roots of *Eurycoma longifolia*. J. Nat. Prod..

[77] Miyake K., Tezuka Y., Awale S., Li F., Kadota S. (2010). Canthin-6-one alkaloids and a tirucallanoid from *Eurycoma longifolia* and their cytotoxic activity against a human HT-1080 fibrosarcoma cell line. Nat. Prod. Commun..

[78] Lin L.C., Peng C.Y., Wang H.S., Lee K.W., Wang P.S. (2001). Reinvestigation of the chemical constituents of *Eurycoma longifolia*. Chin. Pharm. J..

[79] Souza-Almeida E.S., Niero R., Clasen B.K., Balogun S.O., Oliveira-Martins D.T. (2011). Pharmacological mechanisms underlying the anti-ulcer activity of methanol extract and canthin-6-one of *Simaba ferruginea* A. St-Hil. in animal models. J. Ethnopharmacol..

[80] Donkwe S.M.M., Happi E.N., Wansi J.D., Lenta B.N., Devkota K.P., Neumann B., Stammler H.-G., Sewald N. (2012). Oxidative Burst Inhibitory and Cytotoxic Activity of Constituents of the Fruits of *Odyendyea gabonensis*. Planta Med..

[81] Jiang M.X., Zhou Y.J. (2008). Canthin-6-one alkaloids from *Picrasma quassioides* and their cytotoxic activity. J. Asian Nat. Prod. Res..

[82] Varghese C., Ambrose C., Jin S., Lim Y., Keisaban T. (2013). Antioxidant and anti-inflammatory activity of *Eurycoma longifolia* Jack. A traditional medicinal plant in Malaysia. Int. J. Pharm. Sci. Nanotechnol..

[83] Morimoto Y., Iwai T., Yoshimura T., Kinoshita T. (1998). Diastereoselective two-directional synthesis and cation transport ability of the central tristetrahydrofuranyl unit of meso polyether glabrescol as naturally occurring podand. Bioorg. Med. Chem. Lett..

[84] Hioki H., Yoshio S., Motosue M., Oshita Y., Nakamura Y., Mishima D., Fukuyama Y., Kodama M., Ueda K., Katsu T. (2004). Enantioselective Total Synthesis of Eurylene, 14-Deacetyl Eurylene, and Their 11-Epimers: The Relation between Ionophoric Nature and Cytotoxicity. Org. Lett..

[85] Oei-Koch A., Kraus L. (1978). Inhaltsstoffe von *Eurycoma longifolia* Jack. I. Sterols, saponine. Plant Med.

[86] Teh C.H., Abdulghani M., Morita H., Shiro M., Hussin A.H., Chan K.L. (2011). Comparative X-Ray and Conformational Analysis of a New Crystal of 13α,21-Dihydroeurycomanone with Eurycomanone from *Eurycoma longifolia* and Their Anti-Estrogenic Activity Using the Uterotrophic Assay. Planta Med..

[87] Siregar L.A.M., Keng C.L., Lim B.P. (2009). Effects of medium constituents on growth and canthinone accumulation in cell suspension cultures of *Eurycoma longifolia* Jack. HAYATI J. Biosci..

[88] Mahmud Siregar L.A., Peng-Lim B., Lai-Keng C. (2004). Effect of cell source and pH of culture medium on the production of canthin-6-one alkaloids from the cell cultures of Tongkat Ali (*Eurycoma longifolia* Jack). J. Plant Biotechnol..

[89] Maziah M., Rosli N., (Methods in Molecular Biology) Saxena P.K., Jain S.M. (2009). The Production of 9-methoxycanthin-6-one from Callus Cultures of (*Eurycoma longifolia* Jack) Tongkat Ali. Protocols for In Vitro Cultures and Secondary Metabolite Analysis of Aromatic and Medicinal Plants.

[90] Kuo P.C., Damu A.G., Wu T.S. (2003). Characterization of the water soluble fraction from the root extract of Eurycoma longifolia. Chin. Pharm. J..

[91] Asiah O., Nurhanan M., Mohd Ilham A. (2007). Determination of bioactive peptide (4.3 kDa) as an aphrodisiac marker in six Malaysian plants. J. Trop. For. Sci..

[92] Lugnataweepon I., Pleuktivorapongkul A., Sirithunyalug J., Leesawat P., Charumanee S., Yotsawimonwat S. (2011). Effects of herbal powder composition on flow and compaction properties. Proceedings of the Kasetsart University Annual Conference.

[93] Rauh M., Groschl M., Rascher W. (2007). Simultaneous quantification of ghrelin and desacyl-ghrelin by liquid chromatography-tandem mass spectrometry in plasma, serum, and cell supernatants. Clin. Chem..

[94] Tareke E., Bowyer J.F., Doerge D.R. (2007). Quantification of rat brain neurotransmitters and metabolites using liquid chromatography/electrospray tandem mass spectrometry and comparison with liquid chromatography/electrochemical detection. Rapid Commun. Mass Spectrom..

[95] Biesaga M., Pyrzynska K. (2009). Liquid chromatography/tandem mass spectrometry studies of the phenolic compounds in honey. J. Chromatogr..

[96] Canabate-Diaz B., Segura Carretero A., Fernandez-Gutierrez A., Belmonte Vega A., Garrido Frenich A., Martínez Vidal J., Duran Martos J. (2007). Separation and determination of sterols in olive oil by HPLC-MS. Food Chem..

[97] Fabre N., Rustan I., de Hoffmann E., Quetin-Leclercq J. (2001). Determination of flavone, flavonol, and flavanone aglycones by negative ion liquid chromatography electrospray ion trap mass spectrometry. J. Am. Soc. Mass Spectrom..

[98] Guo Z., Vangapandu S., Sindelar R., Walker L., Sindelar R. (2005). Biologically active quassinoids and their chemistry: Potential leads for drug design. Curr. Med. Chem..

[99] Curcino Vieira I.J., Braz-Felho R. (2006). Quassinoids: Structural diversity, biological activity and synthetic studies. Stud. Nat. Prod. Chem..

[100] Tan S., Yuen K.H., Chan K.L. (2002). HPLC analysis of plasma 9-methoxycanthin-6-one from *Eurycoma longifolia* and its application in a bioavailability/pharmacokinetic study. Planta Med..

[101] Teh C.H., Murugaiyah V., Chan K.L. (2011). Developing a validated liquid chromatography-mass spectrometric method for the simultaneous analysis of five bioactive quassinoid markers for the standardization of manufactured batches of *Eurycoma longifolia* Jack extract as antimalarial medicaments. J. Chromatogr..

[102] Han Y.M., Jang M., Kim I.S., Kim S.H., Yoo H.H. (2015). Simultaneous quantitation of six major quassinoids in Tongkat Ali dietary supplements by liquid chromatography with tandem mass spectrometry. J. Sep. Sci..

[103] Said M.M., Gibbons S., Moffat A.C., Zloh M. (2014). Rapid detection of sildenafil analogue in *Eurycoma longifolia* products using a new two-tier procedure of the near infrared (NIR) spectra database. Food Chem..

[104] Sharlip I.D., Jarow J.P., Belker A.M., Lipshultz L.I., Sigman M., Thomas A.J., Schlegel P.N., Howards S.S., Nehra A., Damewood M.D. (2002). Best practice policies for male infertility. Fertil. Steril..

[105] Brugh V.M., Lipshultz L.I. (2004). Male factor infertility: Evaluation and management. Med. Clin. N. Am..

[106] Hirsh A. (2003). Male subfertility. BMJ.

[107] Mahdi A., Bano F., Singh R., Singh R., Siddiqui M., Hasan M. (1999). Seminal plasma superoxide dismutase and catalase activities in infertile men. Med. Sci. Res..

[108] Cooper T.G., Noonan E., Von Eckardstein S., Auger J., Baker H.G., Behre H.M., Haugen T.B., Kruger T., Wang C., Mbizvo M.T. (2010). World Health Organization reference values for human semen characteristics. Hum. Reprod. Update.

[109] Bano F., Singh R., Singh R., Siddiqui M., Mahdi A. (1999). Seminal plasma lipid peroxide levels in infertile men. J. Endocrinol. Reprod..

[110] Sikka S.C. (2001). Relative impact of oxidative stress on male reproductive function. Curr. Med. Chem..

[111] Low B.S., Das P.K., Chan K.L. (2013). Standardized quassinoid-rich *Eurycoma longifolia* extract improved spermatogenesis and fertility in male rats via the hypothalamic-pituitary-gonadal axis. J. Ethnopharmacol..

[112] Low B.S., Choi S.B., Wahab H.A., Das P.K., Chan K.L. (2013). Eurycomanone, the major quassinoid in *Eurycoma longifolia* root extract increases spermatogenesis by inhibiting the activity of phosphodiesterase and aromatase in steroidogenesis. J. Ethnopharmacol..

[113] Chen Y., Phang W.M., Mu A.K.W., Chan C.K., Low B.S., Sasidharan S., Chan K.L. (2015). Decreased expression of alpha-2-HS glycoprotein in the sera of rats treated with *Eurycoma longifolia* extract. Front. Pharmacol..

[114] Ismail S.B., Wan Mohammad W.M.Z., George A., Nik Hussain N.H., Musthapa Kamal Z.M., Liske E. (2012). Randomized clinical trial on the Use of PHYSTA freeze-dried water extract of *Eurycoma longifolia* for the improvement of quality of life and sexual well-being in Men. Evid. Based Complement. Altern. Med..

[115] Chan K.L., Low B.S., Teh C.H., Das P.K. (2009). The effect of *Eurycoma longifolia* on sperm quality of male rats. Nat. Prod. Commun..

[116] Ang H., Ngai T. (2001). Aphrodisiac evaluation in non-copulator male rats after chronic administration of *Eurycoma longifolia* Jack. Fundam. Clin. Pharmacol..

[117] Chen C.K., Mohamad W.M.Z.W., Ooi F.K., Ismail S.B., Abdullah M.R., George A. (2014). Supplementation of *Eurycoma longifolia* Jack Extract for 6 Weeks Does Not Affect Urinary Testosterone: Epitestosterone Ratio, Liver and Renal Functions in Male Recreational Athletes. Int. J. Prev. Med..

[118] Ang H., Ngai T., Tan T. (2003). Effects of *Eurycoma longifolia* Jack on sexual qualities in middle aged male rats. Phytomedicine.

[119] Wahab N.A., Mokhtar N.M., Halim W.N.H.A., Das S. (2010). The effect of *Eurycoma longifolia* Jack on spermatogenesis in estrogen-treated rats. Clinics.

[120] Tambi M., Imran M.K. (2010). *Eurycoma longifolia* Jack in managing idiopathic male infertility. Asian J. Androl..

[121] Erasmus N., Solomon M., Fortuin K., Henkel R. (2012). Effect of *Eurycoma longifolia* Jack (Tongkat ali) extract on human spermatozoa *in vitro*. Andrologia.

[122] Noor M.M., Nor A.H.S.M., Hassan L.C. (2004). The effect of *Eurycoma longifolia* Jack (Tongkat Ali) on sexual behaviour and sperm quality in rats. Malays. J. Pharm. Sci..

[123] Mohd Effendy N., Mohamed N., Muhammad N., Naina Mohamad I., Shuid A.N. *Eurycoma longifolia*: Medicinal plant in the prevention and treatment of male osteoporosis due to androgen deficiency. Evid. Based Complement. Altern. Med..

[124] Solomon M., Erasmus N., Henkel R. (2014). *In vivo* effects of *Eurycoma longifolia* Jack (Tongkat Ali) extract on reproductive functions in the rat. Andrologia.

[125] Tambi M., Imran M., Henkel R. (2012). Standardised water-soluble extract of *Eurycoma longifolia*, Tongkat ali, as testosterone booster for managing men with late-onset hypogonadism?. Andrologia.

[126] George A., Henkel R. (2014). Phytoandrogenic properties of *Eurycoma longifolia* as natural alternative to testosterone replacement therapy. Andrologia.

[127] Zanoli P., Zavatti M., Montanari C., Baraldi M. (2009). Influence of Eurycoma longifolia on the copulatory activity of sexually sluggish and impotent male rats. J. Ethnopharmacol..

[128] Ang H.H., Sim M.K. (1998). *Eurycoma longifolia* increases sexual motivation in sexually naive male rats. Arch. Pharm. Res..

[129] Qinna N., Taha H., Matalka K., Badwan A. (2009). A new herbal combination, Etana, for enhancing erectile function: An efficacy and safety study in animals. Int. J. Impot. Res..

[130] Frydrychova S., Opletal L., Macakova K., Lustykova A., Rozkot M., Lipensky J. (2011). Effects of herbal preparation on libido and semen quality in boars. Reprod. Domest. Anim..

[131] Kotirum S., Ismail S.B., Chaiyakunapruk N. (2015). Efficacy of Tongkat Ali (*Eurycoma longifolia*) on erectile function improvement: Systematic review and meta-analysis of randomized controlled trials. Complement. Ther. Med..

[132] Henkel R.R., Wang R., Bassett S.H., Chen T., Liu N., Zhu Y., Tambi M.I. (2014). Tongkat Ali as a potential herbal supplement for physically active male and female seniors—A pilot study. Phytother. Res..

[133] Nadjm B., Behrens R.H. (2012). Malaria: An Update for Physicians. Infect. Dis. Clin. North Am..

[134] World Health Organization (2015). “World Malaria Report: 2012. Geneva: WHO, 2012”. Fecha Consult..

[135] Taylor W.R., Hanson J., Turner G.D., White N.J., Dondorp A.M. (2012). Respiratory Manifestations of Malaria Lung in Malaria. Chest J..

[136] MAR M.R., Noor Rain A., Zhari I., Zakiah I. (2005). Effect of *Eurycoma longifolia* extract on the Glutathione level in Plasmodium falciparum infected erythrocytes *in vitro*. Trop. Biomed..

[137] Cancer Statistics? Cancer Research UK. http://www.cancerresearchuk.org/health-professional/cancer-statistics.

[138] Rubinstein L., Shoemaker R., Paull K., Simon R., Tosini S., Skehan P., Scudiero D., Monks A., Boyd M. (1990). Comparison of *in vitro* anticancer-drug-screening data generated with a tetrazolium assay *versus* a protein assay against a diverse panel of human tumor cell lines. J. Natl. Cancer Inst..

[139] Ito J., Chang F.R., Wang H.K., Park Y.K., Ikegaki M., Kilgore N., Lee K.H. (2001). Anti-AIDS agents. 48. 1 Anti-HIV activity of moronic acid derivatives and the new melliferone-related triterpenoid isolated from Brazilian propolis. J. Nat. Prod..

[140] Morita H., Kishi E., Takeya K., Itokawa H., Tanaka O. (1990). New quassinoids from the roots of *Eurycoma longifolia*. Chem. Lett..

[141] Mahfudh N. Eurycomanone exert antiproliferative activity via apoptosis in hela cells. Proceedings of the International Conference on Mathematics and Natural sciences (ICMNS).

[142] Tong K.L., Chan K.L., AbuBakar S., Low B.S., Ma H.Q., Wong P.F. (2015). The *In Vitro* and *In Vivo* Anti-Cancer Activities of a Standardized Quassinoids Composition from Eurycoma longifolia on LNCaP Human Prostate Cancer Cells. PLoS ONE.

[143] Hajjouli S., Chateauvieux S., Teiten M.H., Orlikova B., Schumacher M., Dicato M., Choo C.Y., Diederich M. (2014). Eurycomanone and Eurycomanol from *Eurycoma longifolia* Jack as Regulators of Signaling Pathways Involved in Proliferation, Cell Death and Inflammation. Molecules.

[144] Ohishi K., Toume K., Arai M.A., Koyano T., Kowithayakorn T., Mizoguchi T., Itoh M., Ishibashi M. (2015). 9-Hydroxycanthin-6-one, a β-Carboline Alkaloid from *Eurycoma longifolia*, Is the First Wnt Signal Inhibitor through Activation of Glycogen Synthase Kinase 3β without Depending on Casein Kinase 1α. J. Nat. Prod..

[145] Pear W.S., Miller J.P., Xu L., Pui J.C., Soffer B., Quackenbush R.C., Pendergast A.M., Bronson R., Aster J.C., Scott M.L. (1998). Efficient and rapid induction of a chronic myelogenous leukemia-like myeloproliferative disease in mice receiving P210 bcr/abl-transduced bone marrow. Blood.

[146] O’Brien S., Berman E., Devetten M., Network N.C.C. (2010). NCCN Clinical Practice Guidelines in Oncology: Chronic Myelogenous Leukemia. http://www.nccn.org/professionals/physician_gls/f_guidelines.asp.

[147] Kim D.W., Goh Y.T., Hsiao H.H., Caguioa P.B., Kim D., Kim W.S., Saikia T., Agrawal S., Roy A., Dai D. (2009). Clinical profile of dasatinib in Asian and non-Asian patients with chronic myeloid leukemia. Int. J. Hematol..

[148] Druker B.J. (2002). STI571 (Gleevec™) as a paradigm for cancer therapy. Trends Mol. Med..

[149] Deininger M.W., Goldman J.M., Melo J.V. (2000). The molecular biology of chronic myeloid leukemia. Blood.

[150] Al-Salahi O.S.A., Ji D., Majid A.M.S.A., Kit-Lam C., Abdullah W.Z., Zaki A., Din S.K.K.J., Yusoff N.M., Majid A.S.A. (2014). Anti-tumor activity of *Eurycoma longifolia* root extracts against K-562 cell line: *In vitro* and *in vivo* study. PLoS ONE.

[151] Tee T.T., Cheah Y.H., Hawariah L.P.A. (2007). F16, a fraction from *Eurycoma longifolia* jack extract, induces apoptosis via a caspase-9-independent manner in MCF-7 cells. Anticancer Res..

[152] Tee T.T., Azimahtol H.L.P. (2005). Induction of apoptosis by *Eurycoma longifolia* Jack extracts. Anticancer Res..

[153] Chung A.S., Ferrara N. (2011). Developmental and pathological angiogenesis. Annu. Rev. Cell. Dev. Biol..

[154] Herbert S.P., Stainier D.Y. (2011). Molecular control of endothelial cell behaviour during blood vessel morphogenesis. Nat. Rev. Mol. Cell Boil..

[155] Goel S., Duda D.G., Xu L., Munn L.L., Boucher Y., Fukumura D., Jain R.K. (2011). Normalization of the vasculature for treatment of cancer and other diseases. Physiol. Rev..

[156] Frater J.L., Kay N.E., Goolsby C.L., Crawford S.E., Dewald G.W., Peterson L.C. (2008). Dysregulated angiogenesis in B-chronic lymphocytic leukemia: Morphologic, immunohistochemical, and flow cytometric evidence. Diagn. Pathol..

[157] Cardenas C., Quesada A.R., Medina M.A. (2011). Anti-angiogenic and anti-inflammatory properties of kahweol, a coffee diterpene. PLoS ONE.

[158] Al-Salahi O.S.A., Kit-Lam C., Majid A.M.S.A., Al-Suede F.S.R., Mohammed Saghir S.A., Abdullah W.Z., Ahamed M.B.K., Yusoff N.M. (2013). Anti-angiogenic quassinoid-rich fraction from *Eurycoma longifolia* modulates endothelial cell function. Microvasc. Res..

[159] Al-Salahi O.S.A., Zaki A.H., Chan K.L., Shah A.M., Al-Hassan F., Abdullah W.Z., Yusoff N.M. (2013). *In vitro* Anti-proliferative and Apoptotic Activities of *Eurycoma longifolia* Jack (Simaroubaceae) on HL-60 Cell Line. Trop. J. Pharm. Res..

[160] Nurhanan M., Hawariah L., Ilham A.M., Shukri M. (2005). Cytotoxic effects of the root extracts of *Eurycoma longifolia* Jack. Phytother. Res..

[161] Razak M.F.A., Aidoo K.E., Candlish A.G. (2007). Mutagenic and cytotoxic properties of three herbal plants from Southeast Asia. Trop. Biomed..

[162] Farouk A.E., Benafri A. (2007). Antibacterial activity of *Eurycoma longifolia* Jack. A Malaysian medicinal plant. Saudi Med. J..

[163] Farouk A., Nawi M., Hassan S. (2008). Antibacterial peptides from *Euycoma longifolia* (Tongkat Ali) and *Labisia pumila* (Kacip Fatimah) leaves in Malaysia. Sci. Brun.

[164] Kong C., Yehye W.A., Rahman N.A., Tan M.W., Nathan S. (2014). Discovery of potential anti-infectives against Staphylococcus aureus using a Caenorhabditis elegans infection model. BMC Complement. Altern. Med..

[165] Hai Dang N., Choo Y.Y., Tien Dat N., Hoai Nam N., Van Minh C., Lee J.H. (2015). 7-Methoxy-(9*H*-β-Carbolin-1-il)-(*E*)-1-Propenoic Acid, a β-Carboline Alkaloid From *Eurycoma longifolia*, Exhibits Anti-Inflammatory Effects by Activating the Nrf2/Heme Oxygenase-1 Pathway. J. Cell. Biochem..

[166] Ang H.H., Cheang H.S. (1999). Studies on the anxiolytic activity of *Eurycoma longifolia* Jack roots in mice. Jpn. J. Pharmacol..

[167] Talbott S.M., Talbott J.A., George A., Pugh M. (2013). Effect of Tongkat Ali on stress hormones and psychological mood state in moderately stressed. J. Int. Soc. Sports Nutr..

[168] Husen R., Pihie A.H.L., Nallappan M. (2004). Screening for antihyperglycaemic activity in several local herbs of Malaysia. J. Ethnopharmacol..

[169] Lahrita L., Kato E., Kawabata J. (2015). Uncovering potential of Indonesian medicinal plants on glucose uptake enhancement and lipid suppression in 3T3-L1 adipocytes. J. Ethnopharmacol..

[170] Kamel H.K. (2005). Male Osteoporosis. Drugs Aging.

[171] Melton L.J., Atkinson E.J., O'Connor M.K., O'Fallon W.M., Riggs B.L. (1998). Bone density and fracture risk in men. J. Bone Miner. Res..

[172] Melton L.J., Chrischilles E.A., Cooper C., Lane A.W., Riggs B.L. (2005). How many women have osteoporosis?. J. Bone Miner. Res..

[173] Kanis J., Johnell O., Oden A., Sernbo I., Redlund-Johnell I., Dawson A., De Laet C., Jonsson B. (2000). Long-term risk of osteoporotic fracture in Malmö. Osteoporos. Int..

[174] Shuid A.N., Abu Bakar M.F., Abdul Shukor T.A., Muhammad N., Mohamed N., Soelaiman I.N. (2011). The anti-osteoporotic effect of *Eurycoma longifolia* in aged orchidectomised rat model. Aging Male.

[175] Ali J., Saad J. (1993). Biochemical Effect of *Eurycoma longifolia* Jack on the Sexual Behavior, Fertility, Sex Hormone, and Glycolysis. Ph.D. Thesis.

[176] Hooi Hoon A., Cheang H.S., Yusof A.P.M. (2000). Effects of *Eurycoma longifolia* Jack (Tongkat Ali) on the initiation of sexual performance of inexperienced castrated male rats. Exp. Anim..

[177] Moreira S.G., Brannigan R.E., Spitz A., Orejuela F.J., Lipshultz L.I., Kim E.D. (2000). Side-effect profile of sildenafil citrate (Viagra) in clinical practice. Urology.

[178] Sahelian R. (2003). Natural Sex Boosters: Supplements That Enhance Stamina, Sensation, and Sexuality for Men and Women.

[179] Halliwell B., Gutteridge J.M. (1999). Free Radicals in Biology and Medicine.

[180] Wauquier F., Leotoing L., Coxam V., Guicheux J., Wittrant Y. (2009). Oxidative stress in bone remodelling and disease. Trends Mol. Med..

[181] Saadiah Abdul Razak H., Shuid A.N., Naina Mohamed I. (2012). Combined effects of *Eurycoma longifolia* and testosterone on androgen-deficient osteoporosis in a male rat model. Evid. Based Complement. Altern. Med..

[182] Shuid A.N., El-arabi E., Effendy N.M., Razak H.S.A., Muhammad N., Mohamed N., Soelaiman I.N. (2012). *Eurycoma longifolia* upregulates osteoprotegerin gene expression in androgen-deficient osteoporosis rat model. BMC Complement. Altern. Med..

[183] Abdulghani M., Hussin A.H., Sulaiman S.A., Chan K.L. (2012). The ameliorative effects of *Eurycoma longifolia* Jack on testosterone-induced reproductive disorders in female rats. Reprod. Biol..

[184] Muhamad A.S., Keong C.C., Kiew O.F., Abdullah M.R. (2009). *Eurycoma longifolia* Jack: Medicinal properties and its effect on endurance exercise performance. Asian J. Exerc. Sports Sc..

[185] Ulbricht C., Conquer J., Flanagan K., Isaac R., Rusie E., Windsor R.C. (2013). An Evidence-Based Systematic Review of Tongkat Ali (*Eurycoma longifolia*) by the Natural Standard Research Collaboration. J. Diet. Suppl..

[186] Jantan I., Zaki Z., Ahmad A., Ahmad R. (1999). Evaluation of smoke from mosquito coils containing Malaysian plants against Aedes aegypti. Fitoterapia.

[187] Girish S., Kumar S., Aminudin N. (2015). Tongkat Ali (*Eurycoma longifolia*): A possible therapeutic candidate against Blastocystis sp. Parasites Vectors.

[188] Ang H.H., Cheang H.S. (2001). Effects of Eurycoma longifolia Jack on laevator ani muscle in both uncastrated and Testosterone-Stimulated castrated intact male rats. Arch. Pharmacal Res..

[189] Qodriyah H., Asmadi A. (2013). *Eurycoma longifolia* in Radix (TM) for the Treatment of Ethanol-induced Gastric Lesion in Rats. Pak. J. Biol. Sci..

[190] Bich D., Chung D., Chuong B., Dong N., Dam D., Hien P., Lo V., Mai P., Man P., Nhu D. (2004). The medicinal plants and animals in Vietnam. Hanoi Sci. Technol. Publ. House Hanoi.

[191] Chan K.L., Low B.S., San Ho D.S. (2010). Polar Organic Extract of Eurycoma longifolia. U.S. Patent.

[192] Pan Y., Tiong K.H., Abd-Rashid B.A., Ismail Z., Ismail R., Mak J.W., Ong C.E. (2014). Effect of eurycomanone on cytochrome P450 isoforms CYP1A2, CYP2A6, CYP2C8, CYP2C9, CYP2C19, CYP2E1 and CYP3A4 *in vitro*. J. Nat. Med..

[193] Han Y.M., Kim I.S., Rehman S.U., Choe K., Yoo H.H. (2015). In Vitro Evaluation of the Effects of *Eurycoma longifolia* Extract on CYP-Mediated Drug Metabolism. Evid. Based Complement. Altern. Med..

[194] Satayavivad J., Noppamas S., Aimon S., Yodhathai T. (1998). Toxicological and antimalaria activity of *Eurycoma longifolia* Jack extracts in mice. Thai J. Phytopharm..

[195] Shuid A., Siang L., Chin T., Muhammad N., Mohamed N., Soelaiman I. (2011). Acute and Subacute Toxicity Studies of *Eurycoma longifolia* in Male Rats. Int. J. Pharm..

[196] Choudhary Y.K., Bommu P., Ming Y.K., Zulkawi N.B. (2012). Acute, sub-acute, and subchronic 90-days toxicity of *Eurycoma longifolia* aqueous extract (Physta) in wistar rats. Int. J. Pharm. Pharm. Sci..

[197] Bhasin S., Cunningham G.R., Hayes F.J., Matsumoto A.M., Snyder P.J., Swerdloff R.S., Montori V.M. (2010). Testosterone therapy in men with androgen deficiency syndromes: An Endocrine Society clinical practice guideline. J. Clin. Endocrinol. Metab..

[198] Li C.H., Liao J.W., Liao P.L., Huang W.K., Tse L.S., Lin C.H., Kang J.J., Cheng Y.W. (2013). Evaluation of Acute 13-Week Subchronic Toxicity and Genotoxicity of the Powdered Root of Tongkat Ali (*Eurycoma longifolia* Jack). Evid. Based Complement. Altern. Med..

[199] Hamoud H., Qamar U. (2013). Effect of long-term use of *Eurycoma longifolia* Jack on the pancreas in rats. Histol. Assess..

[200] Low B.S., Das P.K., Chan K.L. (2014). Acute, Reproductive Toxicity and Two-generation Teratology Studies of a Standardized Quassinoid-rich Extract of *Eurycoma longifolia* Jack in Sprague-Dawley Rats. Phytother. Res..

[201] Food and Drug Administration Guidance for Industry, Estimating the Maximum Safe Starting Dose in Initial Critical Trials for Therapeutics in Adult Healthy Volunteers. http://www.fda.gov/downloads/Drugs/.../Guidances/UCM078932.pdf.

[202] Salman S., Amrah S., Wahab M., Ismail Z., Ismail R., Yuen K., Gan S. (2010). Modification of propranolol’s bioavailability by *Eurycoma longifolia* water-based extract. J. Clin. Pharm. Ther..

[203] Jellin J.M., Batz F., Hitchens K. (2016). Natural Medicines Comprehensive Database.

[204] Bramwell D. (2002). How many plant species are there?. Plant Talk.

[205] Verpoorte R., van der Heijden R., Memelink J. (2000). Engineering the plant cell factory for secondary metabolite production. Transgenic Res..

[206] Wolfender J.L., Ndjoko K., Hostettmann K. (2003). Liquid chromatography with ultraviolet absorbance-mass spectrometric detection and with nuclear magnetic resonance spectrometry: A powerful combination for the on-line structural investigation of plant metabolites. J. Chromatogr..

[207] Vuorela P., Leinonen M., Saikku P., Tammela P., Wennberg T., Vuorela H. (2004). Natural products in the process of finding new drug candidates. Curr. Med. Chem..

